# Toxicological Assessment of Cellulose Nanomaterials: Oral Exposure

**DOI:** 10.3390/nano12193375

**Published:** 2022-09-27

**Authors:** Nádia Vital, Célia Ventura, Michel Kranendonk, Maria João Silva, Henriqueta Louro

**Affiliations:** 1 National Institute of Health Dr. Ricardo Jorge, Department of Human Genetics, 1649-016 Lisbon, Portugal; 2 NOVA Medical School, Universidade NOVA de Lisboa, 1169-056 Lisbon, Portugal; 3 Centre for Toxicogenomics and Human Health (ToxOmics), NOVA Medical School, Universidade NOVA de Lisboa, 1169-056 Lisbon, Portugal

**Keywords:** cellulose nanomaterials, cellulose nanocrystals, cellulose nanofibres, ingestion, biological effects, nanotoxicology

## Abstract

Cellulose nanomaterials (CNMs) have emerged recently as an important group of sustainable bio-based nanomaterials (NMs) with potential applications in multiple sectors, including the food, food packaging, and biomedical fields. The widening of these applications leads to increased human oral exposure to these NMs and, potentially, to adverse health outcomes. Presently, the potential hazards regarding oral exposure to CNMs are insufficiently characterised. There is a need to understand and manage the potential adverse effects that might result from the ingestion of CNMs before products using CNMs reach commercialisation. This work reviews the potential applications of CNMs in the food and biomedical sectors along with the existing toxicological in vitro and in vivo studies, while also identifying current knowledge gaps. Relevant considerations when performing toxicological studies following oral exposure to CNMs are highlighted. An increasing number of studies have been published in the last years, overall showing that ingested CNMs are not toxic to the gastrointestinal tract (GIT), suggestive of the biocompatibility of the majority of the tested CNMs. However, in vitro and in vivo genotoxicity studies, as well as long-term carcinogenic or reproductive toxicity studies, are not yet available. These studies are needed to support a wider use of CNMs in applications that can lead to human oral ingestion, thereby promoting a safe and sustainable-by-design approach.

## 1. Introduction

Cellulose nanomaterials (CNMs), also known as nanocelluloses or cellulosic nanomaterials, are defined as “materials composed predominantly of cellulose, with any external dimension in the nanoscale or materials having an internal structure or surface structure in the nanoscale, with the internal structure or surface structure composed predominantly of cellulose” [1]. These bio-based nanomaterials (NMs) are derived from cellulose—an abundant, renewable, and biodegradable organic polymer found mostly in plants. CNMs are currently being investigated as sustainable/“green” materials in a wide range of different innovative end-user products, such as paper, adhesives and paints, cosmetics, and hygiene, among many others [2,3,4,5,6,7,8]. CNMs have also been intensively studied for applications in the food sector as food additives—for instance, as zero-calorie stabilisers, fillers or thickeners [3,9,10,11], or in food packaging nanocomposites, as bio-based substitutes for petroleum-derived materials, in an effort to reduce plastic usage [12]. Biomedical applications (e.g., as drug excipients or delivery systems, including oral drug delivery systems) are also anticipated [4,5,8,13]. These new developments have been made possible due to the CNMs’ physicochemical characteristics. These include their nanoscale size, fibril morphology, and large surface area [14], leading to high crystallinity, excellent mechanical strength, stiffness, and low weight. Moreover, CNMs have demonstrated the capacity to produce two-dimensional (2D) and three-dimensional (3D) structures, including films, membranes, foams, and porous aerogels, among others [4]. Thus, CNMs can bring additional advantages to current products, including improved mechanical and barrier properties and increased recyclability, among others [12,15,16,17]. Most of these applications are at the development stage, with production at the pilot scale [18,19,20]. Nevertheless, the global CNM market is projected to grow from USD 346 million in 2021 to USD 963 million by 2026 [21]. Thus, the production of CNMs is anticipated to have a major economic impact. CNMs have great potential in replacing other resource-intensive materials—such as plastics from fossil fuels—and from a circular economy perspective, since they can be obtained from waste from various industrial sources. The foreseen expansion of the production and applications of CNMs is expected to lead to increased environmental exposure throughout products’ lifecycles; thus, early identification of relevant exposure scenarios for the manufacture, use, and disposal of CNMs is needed to minimise those potential issues. Moreover, increased human exposure is also foreseen—both at the workplace (e.g., in production plants) and through consumers’ products or medical procedures. Addressing the potential impact of these materials on human health and the environment is necessary before their large-scale production and commercialisation, as an integral part of the innovation process, for their safe and sustainable development [22,23].

Due to their natural origin, cellulose-based materials are often assumed to be biocompatible and non-toxic [4,24]. However, their properties at the nanoscale may lead to more reactive and potentially toxic materials. The toxicity of CNMs has been reviewed recently by different authors, but most studies have focused mainly on the respiratory tract/inhalation route of exposure [15,18,22,23,24,25,26,27,28,29,30,31]. The knowledge of the toxicity of CNMs via this route has been seen as a priority, mostly due to potential occupational exposure in production or processing facilities [15,29]. Additionally, bio-persistent high-aspect-ratio nanomaterials (HARNs) have been associated with adverse biological effects that may lead to lung disease given the fibre pathogenicity paradigm [24,28]. Still, evidence of CNMs fitting the fibre paradigm is lacking [29,32]. Moreover, the safety assessment of respiratory exposure to CNMs has shown discordant results [22,23]. The safety assessment of exposure to CNMs via other routes, such as dermal contact or ingestion, has been less explored. The latter is of relevance, since human exposure to CNMs is expected to increase—either directly, through the ingestion of food products or pharmaceutical drugs containing CNMs, or indirectly, through food contaminated with CNMs released from food packaging materials [15]. Therefore, the present paper provides a review of the studies on the safety assessment of new CNMs with potential applications in food, food packaging, or oral pharmaceuticals that may impact the gastrointestinal tract (GIT). The aim was to identify potential toxic effects, if any, triggered after the ingestion of CNMs. Knowledge gaps and key aspects to be considered are highlighted, contributing to the design of future studies, and to a safe and sustainable-by-design (SSBD) approach in the development of CNMs, to support their safe use in end products. For this purpose, this work provides (i) a brief review of the types and applications of CNMs in food technology and biomedicine; (ii) an overview of the interactions of CNMs with food digestion and possible effects on CNMs’ properties and food components; (iii) a comprehensive review of the literature reporting their biological impact, along with their specific physicochemical characteristics; and (iv) an overview of relevant features to be considered in the toxicity assessment of CNMs.

## 2. Production and Application of CNMs

### 2.1. Overview of Sources and Production of CNMs

Cellulose is a white, fibre-like structure consisting of a linear polysaccharide chain composed of repeated units of two D-glucopyranose rings linked together by oxygen covalently bonded to C1 of one glucose ring and C4 of the adjoining ring (β-1,4 glycosidic bond), with free hydroxyl groups (-OH) at the C-2, C-3, and C-6 atoms [12,28]. Cellulose materials can be produced at the nanoscale, and the resulting CNMs may be grouped into five main categories based on the cellulose source, extraction/production method, and surface chemistry [14,33]. These categories are cellulose nanocrystals (CNCs), cellulose nanofibrils (CNFs), cellulose nanocrystals from tunicates (t-CNCs), algal cellulose (AC), and bacterial nanocellulose (BC) [14,23,33,34].

**Figure 1:**
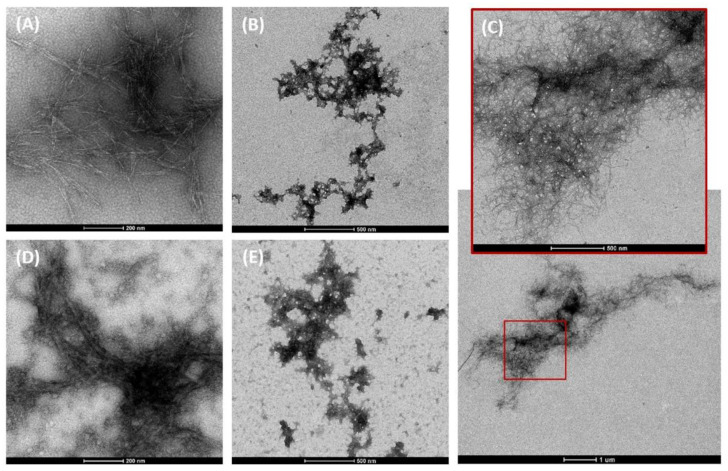
TEM images of the three cellulose nanomaterials dispersed in PBS (**A**–**C**) or complete RPMI cell culture medium (**D**,**E**). (**A**,**D**) CNC obtained by acid hydrolysis; (**B**,**E**) CNF obtained via an enzymatic treatment; (**C**) CNF obtained by TEMPO. Reproduced with permission from [47], *Nanomaterials*, published by MDPI, 2022.

The most common sources of CNMs—particularly of CNFs and CNCs—are cellulose obtained from plants, namely, hardwood or softwood pulp. Plant cellulose can also be extracted from seed fibres, bast fibres, grasses, banana peel, oil palm, and rice straw, among others [13,16,23,35]. Cellulose is predominantly located in the plant’s secondary cell wall, which is reinforced by a matrix also consisting of lignin, hemicellulose, pectin, proteins, extractive organic substances, and trace elements [23,34,36]. Large cellulose fibres are constituted by parallel stacking of multiple cellulose chains that form the elementary fibrils and are assembled into larger microfibril bundles [23,34]. The elementary cellulose fibrils are constituted by regions of highly ordered cellulose chains (i.e., crystalline) that form the core, which alternate with disordered regions (i.e., amorphous) [23,34]. CNFs—also known as nanofibrillated cellulose, nanofibrillar cellulose, or cellulose nanofibres—are defined as “cellulose nanofibres composed of at least one elementary fibril, containing crystalline, paracrystalline and amorphous regions, with aspect ratios usually greater than 10 nm, which may contain longitudinal splits, entanglement between particles, or network-like structures” [1]. CNFs’ dimensions are typically 3–100 nm in cross-section (diameter) and up to 100 μm in length [1]. CNCs—also known as nanocrystalline cellulose, cellulose nanowhiskers, needles, spheres, or nanowires—are defined as “nanocrystals predominantly composed of cellulose with at least one elementary fibril containing predominantly crystalline and paracrystalline regions, with aspect ratio ranging from 5 to 50, not exhibiting longitudinal splits, inter-particle entanglement, or network-like structures” [1]. CNCs’ dimensions are typically 3–50 nm in cross-section and 100 nm to several μm in length, depending on the source material [1]. CNFs or CNCs are obtained by fragmentation of the cellulose hierarchical structure using different chemical, enzymatic, and/or mechanical approaches. An overview of the extraction processes for CNFs and CNCs is detailed elsewhere [36,37,38]. Briefly, the production of CNFs requires breaking the fibres into delaminated individual nanofibrils. These are mainly obtained via high-energy mechanical shearing methods, such as ultrafine grinding/microgrinding, microfluidisation, high-intensity ultrasonication, or high-pressure homogenisation, among others [3,36,38,39]. These processes are normally preceded by chemical or enzymatic hydrolysis treatments to increase the fragmentation/depolymerisation (nanofibrillation) efficiency and reduce production costs [3,29,36,37,39]. These treatments also contribute to removing non-cellulosic constituents such as lignin and hemicelluloses, producing highly purified cellulose, and can yield CNFs with modified surface chemistry [36]. Common chemical treatments leading to chemical modifications of CNFs include catalytic oxidation with 2,2,6,6-tetramethylpiperidine-1-oxyl radicals (TEMPO oxidation), whereby the primary hydroxyl groups on the C6 position of cellulose are converted to carboxylic groups [40,41,42]; carboxymethylation [43,44,45]; phosphorylation [44,45]; etc. CNCs are derived from the crystalline regions of cellulose and are commonly extracted by acid hydrolysis of cellulose pulp using mineral acids—typically sulfuric, hydrochloric, or phosphoric acid [33]. Acid hydrolysis removes non-cellulosic components and annihilates most of the amorphous regions, leaving the crystalline regions, and resulting in the formation of nanocrystal structures [33,34,38,46]. CNMs extracted from different sources via different production methods are analogous in chemical composition but have different morphologies, lengths, widths, aspect ratios, degrees of polymerisation (i.e., the number of glucose units), and crystallinity [3,14,20,34]. Different ratios of amorphous fractions and crystalline domains influence the CNMs’ physical characteristics. While CNCs have a short needle- or rod-like morphology demonstrating similar diameters and a high level of rigidity/stiffness due to their high crystallinity, entangled CNFs have fibre/fibril morphologies with a higher aspect ratio, plasticity, and flexibility [23,33]. Figure 1 presents transmission electron microscopy (TEM) images of a CNC obtained by acid hydrolysis, a CNF obtained via an enzymatic treatment, and a CNF obtained by TEMPO.

The accessibility of hydroxyl groups on the cellulose surface and the relatively large specific surface area of CNMs offer many possibilities for their modification and functionalisation during production, resulting in different surface chemistries [4,14,33,39]. Chemical modifications can occur as a byproduct of the extraction process (e.g., sulphate half-ester formation after treatment with sulfuric acid, carboxylic acid after treatment with TEMPO, etc.) [48]. Surface functionalisation can also occur via adsorption to the surface of the particles and covalent attachment of molecules or derivatisation of the surface [48]. Molecular grafting, grafting of polymers or supramolecular units, the addition of fluorescent tags, and nanoparticles, among others, are often used to functionalise CNMs [14,45,48,49,50,51,52]. The variety of chemistries currently being used has been summarised in recent reviews [39,45,48,53,54]. The surface chemistry affects the CNMs’ hydrophilic/hydrophobic balance and its interaction with the surrounding environment, influencing CNMs’ degree of aggregation and agglomeration, dispersibility in solvents or polymers, rheology, and applicability in multiple systems [14,33,39,55,56]. Moreover, and most importantly with respect to the focus of this review, surface chemistry can also influence CNMs’ interactions with biological systems [57,58,59].

### 2.2. CNMs in Food Technology and Biomedicine

Conventional cellulose and some of its derivatives have a long history of use as additives in food and animal feed. In the European Union, several micron-sized or larger celluloses and cellulose derivatives are currently authorised as food additives in almost all food categories, at quantum satis (QS), as defined in Annex II of Regulation (EC) No 1333/2008 on food additives. They are considered safe for use as food additives, and an acceptable daily intake (ADI) has been considered unnecessary, based on their low toxicity, absence of genotoxic concerns and, if any, their negligible absorption in the human GIT [60]. Several celluloses and their derivatives are also authorised under the European Regulation (EC) No 10/2011 for plastics for food packaging, as well as for use as polymer additives, production aids, and other starting substances. Conversely, CNMs have not yet been authorised as food additives or as food contact materials in Europe. Specific assessments are required for their safety evaluation in the framework of food and feed in the European Union, as described by the European Food Safety Authority (EFSA)’s guidance on nanomaterials [61]. In the USA, celluloses have been designated “generally regarded as safe” (GRAS) by the US Food and Drug Administration (FDA) and are approved as food additives by the US Department of Agriculture’s Food Safety and Inspection Service [62,63]. No specific regulatory provisions are in place for pharmaceutical drugs or other medical products and devices using NMs in the USA, Canada, the UK, Japan, or Europe [64]. In Europe, some guidelines are available for specific NMs for applications in human medicines [65,66].

**Figure 2:**
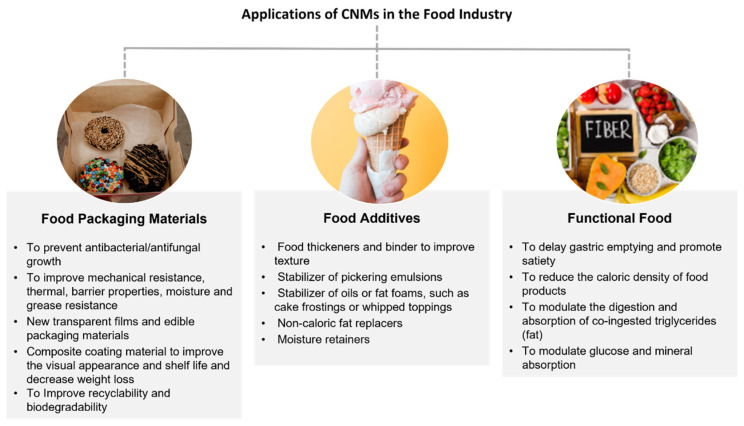
Potential applications of CNMs in the food industry, including food contact materials.

CNMs have found a multitude of potential applications in the food industry [2,46,67,68]. Three main categories of applications can be foreseen: food packaging materials, food additives, and functional foods [46,67], as depicted in Figure 2. It is worth noting that, to the best of our knowledge, food contact materials and foods containing CNMs have not yet reached the market.

There is currently a great demand for new and innovative food packaging materials driven by the policy of using fewer plastics and increasing recyclability. Replacing conventional non-renewable and practically non-biodegradable oil-based materials with CNM-based packaging materials could increase the biodegradability of food packaging and reduce packaging waste, minimising the environmental impact [12,68,69]. Several nanocomposite food packaging materials containing CNMs have been developed, showing promising results in improving the food packaging’s main functions, i.e., extending food’s stability and assuring its quality and safety for a longer shelf-life [2,3,12,16,68,69,70,71,72]. CNMs can be incorporated as reinforcing structures in different types of food packaging materials (e.g., as a filler or coating), to improve transparency or mechanical (e.g., tensile strength), thermal, and barrier (e.g., gases and water vapour) properties, as compared to conventional materials [12,67,68,71,73,74,75]. When applied as coating materials in the inner layer of food packaging materials (such as paper), CNMs can contribute to protecting packaging from water (e.g., cups for coffee) or increasing its resistance to grease/oil (e.g., in pizza/hamburger boxes) [71]. CNMs can also be added to packaging composites to increase anti-microbiological performance in various types of foods and materials [67,71,74,76,77,78]. Additionally, the quality of fruits and vegetables (e.g., strawberries, blueberries, pomegranate seeds, or pears) has been improved when coated with CNM composites (e.g., chitosan/CNMs or CNFs/nanoparticles of calcium carbonate), showing decreased weight loss and improved food preservation, or improved visual appearance [79,80,81,82]. Edible packaging materials made of CNM composites with either increased antimicrobial properties or improved mechanical strength or barrier properties have also been investigated [83,84,85].

As food additives, CNMs have been proposed as stabilisers of oil-in-water Pickering emulsions with or without antibacterial properties, to improve food’s homogeneity and stability [11,86,87,88]. CNMs or CNM composites may be used to stabilise oils or fat foams, such as cake frostings or whipped toppings, or emulsions such as salad dressings, sauces, and gravies [46]. They can also potentially be applied as rheology modifiers—for example, to improve gelling (i.e., anti-melting) and texture properties, as a fat substitute to reduce the caloric value of ice cream [89,90], or to thicken powder-based soy milk [46]. Additionally, they have been proposed as a non-caloric fat substitute in meat sausages using nanocellulose-stabilised soybean oil to retain moisture without compromising texture [91].

One of the main advantages of CNMs is their appeal to dietetic foods, due to their indigestibility by humans. The use of CNMs as functional foods has also been proposed, with their effects being studied in various aspects of the digestion process as a non-caloric fibre source (i.e., dietary fibre) to reduce the caloric density of food products [9]. They can also be used as modulating agents in the digestion and absorption of co-ingested triglycerides (i.e., fat) [10], to delay the digestion and diffusion of starch [92], or to modulate the absorption of glucose and minerals [92,93].

**Figure 3:**
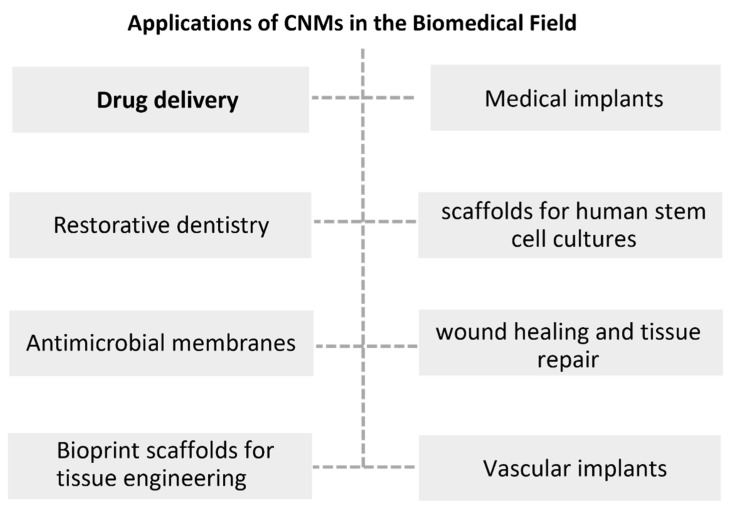
Potential applications of cellulose nanomaterials in biomedicine.

In addition to their uses in the food industry, CNMs have been characterised as very promising materials for biomedical and pharmaceutical applications, as depicted in Figure 3. This is due to their supposed biocompatibility, chemical modification capabilities (vide supra), water-retaining capacity, hydrophilic properties, advantageous mechanical properties (such as high mechanical strength), and relatively inexpensive production. All of these characteristics are paired with their biodegradability, renewability, and ready availability [4,94], enabling different types of formulations, either alone or as polymer composites [94]. Multiple potential applications of CNCs or CNFs in the biomedical field have been explored in recent years, as extensively reviewed in [5,8,13,20,94,95,96,97,98,99,100,101,102]. Among many biomedical applications, CNMs have been widely investigated for the delivery of hydrophilic, hydrophobic, water-soluble, and poorly water-soluble drugs, to be applied via different routes of administration [4,25,95]. CNM-based systems using different carrier forms (for example, dry foams and films, aerogels, emulsions, or hydrogels) have been studied to mediate drug delivery (e.g., riboflavin, bendamustine, hydrochloride, naproxen and ibuprofen, furosemide, methotrexate, repaglinide) for oral administration [94,103,104,105,106,107,108,109]. These systems have been designed for sustained and targeted drug release, with decreased side effects and enhanced therapeutic efficacy over a prolonged period, and with the prospect of dose reduction [104]. Applications of CNMs in bio-adhesive films have also been sought for controlled drug delivery or local drug administration, as shown for the colon-specific delivery of methotrexate [110]. Other applications of CNMs in the biomedical field include restorative dentistry, bioprinting for tissue engineering, wound healing and tissue repair, medical implants, vascular grafts, bone tissue engineering, antimicrobial membranes, and scaffolds for human stem cell cultures, among others [4,5,20,94,97,111,112,113,114,115,116,117].

The majority of applications of CNMs in food technology or biomedical applications are still in the early R&D stages, but they are expected to reach the market in the near future. Nevertheless, as recently suggested, CNMs—and particularly CNCs—might already be present in many food products and pharmaceuticals [118]. Studies have suggested that CNCs may represent a small fraction of the particles present, for example, in microcrystalline cellulose (MCC)—a typical food and drug additive that is considered to be safe. This observation was based on a preliminary study, in which particles with diameter/width less than 100 nm were found after serial filtration of MCC suspensions [118]. However, the presence of CNMs in food/food packaging products or pharmaceutical/nutraceutical drugs is currently largely unknown. All of these expectations raise safety issues regarding the ingestion of CNMs, either from food, edible materials (such as edible films and coatings, or drugs), or migration from food packaging to food components [11,15]. Therefore, it is paramount, as for any NMs used in food technology or oral drug administration, that a thorough assessment is made of the risks that CNMs might pose to human health and the environment [64,119].

## 3. CNMs’ Digestion and Fate in the GIT 

NMs may be ingested by humans and undergo digestion, which acts (together with the mucosal layers) as a selective barrier to systemic particulate exposure. Digestion results in most NMs being fully eliminated from the body via the faeces [120]. Some dietary fibres, if at the nanoscale, may reach the small intestine with their features largely intact, since these fibres are not processed by digestive enzymes in the upper GIT but may be fermented by enzymes released by the microbiota in the lower GIT (colon) [121]. This is expected to be the case for cellulose fibres, such as CNMs. The carbohydrate NMs that are not digested may be absorbed by the body, exerting local effects, or may interact with the gut microbiota, potentially leading to adverse health effects [121]. For example, a recent study on rats demonstrated altered microbial diversity of the GIT when they ingested CNFs, resulting in a reduction in the abundance of specific strains that produce short-chain fatty acids, associated with increased serum insulin and IgA production [122]. These observations highlight the importance of understanding the fate of ingested CNMs along the GIT, as well as the factors involved [123,124]. Currently, knowledge on the bio-persistence or bio-durability of CNMs in the human body is lacking [27]. Proper studies on the uptake and translocation of CNMs by intestinal cells are absent and, to the best of our knowledge, no in vivo toxicokinetic studies on oral exposure to CNMs are available. Still, with the possibility of achieving imaging of CNMs within cells by fluorescent labelling, it is expected that this scenario will soon be changed.

The potential uptake and local effects of nanofibres in the small intestine can be influenced by their physicochemical properties [61]. In vitro digestion models representative of the human gastrointestinal tract have been applied to study the possible effects of the digestion process on the degradation and physicochemical properties of CNMs. These include aspect ratio, morphology, polydispersity, surface charge, surface chemistry, crystallinity index, colloidal stability, and rheological properties. The application of in vitro digestion models has been considered by the EFSA as a key first step in the framework for in vitro and in vivo testing of NMs [61]. Available static in vitro digestion models—such as the standardised INFOGEST in vitro digestion method [125,126], among other models [127]—have been used to study different aspects of NMs’ digestion. These in vitro models simulate the human GIT’s sequential oral, gastric, and intestinal digestion to some extent, along with its physiological conditions. These include the pH, duration of digestion, enzyme concentration and activity, and composition of simulated digestive fluids (including electrolytes and bile) of the upper GIT, namely, the oral, gastric, and small intestinal phases [125]. Although not specific to NMs and not an officially standardised method [61], the EFSA recognises the INFOGEST in vitro digestion method [125,126] as a suitable method to mimic the digestion of NMs under feeding conditions. The applicability of these digestion methods in in vitro toxicological studies can improve their predictability, representing an alternative to animal models and providing more insight into the NMs’ dissolution/degradation rates and degrees of aggregation/agglomeration within the different GIT compartments [128]. For example, the degree of aggregation/agglomeration of several NMs was shown to vary in artificial saliva, gastric fluid, and intestinal juices [129,130,131]. Our previous study showed that an anatase/rutile TiO_2_ mixture (NM-105) displayed more pronounced toxicity to HT29-MTX-E12 intestinal cells after simulated in vitro digestion (without the presence of food), as compared to the undigested NM-105, concomitant with lower hydrodynamic size upon in vitro digestion as compared to undigested samples [132,133]. A non-exhaustive summary of studies that applied in vitro digestion models to address different aspects of CNMs upon digestion is presented in Table 1.

The dispersibility or digestibility of NMs in GIT fluids is known to be influenced by their dimensions, as well by their interaction with the GIT’s components, such as digestive enzymes, bile salts, mineral ions, and phospholipids [92,124]. Considering that CNMs are derived from cellulosic materials, and that cellulose is an insoluble fibre that exhibits a gelling behaviour normally seen in soluble fibres, CNMs may show similar effects to dietary fibre on human digestive health [124]. The particle size of dietary fibre influences its transit time, fermentation rate, and faecal excretion in the digestive tract [124]. Therefore, CNMs with different physicochemical properties can distinctly influence food digestion and nutrient absorption [124]. Moreover, along the GIT, CNMs are exposed to multiple factors that may also play a role in their fate, such as digestive enzymes (including amylases, lipases, and proteases), biopolymers (e.g., mucin), variations in pH and ionic composition, surface-active components (including bile salts, fatty acids, proteins, and phospholipids), or the gastrointestinal flow/movements [124,140]. The application of the digestion model described by Minekus et al. to sulphated CNCs did not indicate any significant changes to their particle size distribution and surface charge based on their hydrodynamic diameter, dispersity index, and zeta potential as they undergo digestion. [138]. This was corroborated by another study where CNFs, CNF–TEMPO, and CNCs demonstrated no significant changes during all digestion phases, except when CNFs and CNF–TEMPO were in the presence of a whey protein isolate, which resulted in a smaller mean particle size at the gastric phase [139]. This is consistent with the current understanding of cellulose metabolism, in which humans lack digestive enzymes capable of breaking down cellulose in the gastrointestinal tract [138]. Nevertheless, upon reaching the colon, the possible digestion or degradation of CNMs by the microbiota can potentially lead to smaller fibres [61]. In this regard, in vitro digestion procedures incorporating microbiota and toxicokinetic (e.g., ADME) studies would be useful. The CNMs’ aggregation/agglomeration state may also play an important role in their fate along the GIT [124]. TEMPO–CNFs formed aggregates, clumps, and phase separation in the gastric phase, while CNCs formed a hydrogel network in the gastric phase, causing increased viscosity [92,139].

CNMs’ agglomeration upon in vitro digestion was also studied by Cao et al. (2020) using the in vitro digestion protocol presented by Deloid et al. (2017). CNCs were digested in a fasting diet model (i.e., FFM; fasting food model) consisting of phosphate buffer or a standardised food model (SFM) based on the average American diet. In the absence of CNCs, a population of smaller particles (<1 μm) was observed with the SFM, while in the presence of CNCs the majority of particles had a size around 20–30 μm in both the SFM and FFM [134]. In the absence of CNCs, the particle size distributions of oral, gastric, and small intestinal phase digestions were similar in all three phases, showing a single broad peak at 3–6 μm and 10–20 μm for the FFM and SFM, respectively. In the presence of CNCs, both digestions had similar distributions, with a single broad peak at 10–20 μm, similar to that of SFM digestions without CNCs [134]. These results suggest that CNCs may interact with digestive proteins (e.g., mucin, pancreatic enzymes, pepsin) and food molecules, forming larger mixed agglomerates during in vitro simulated digestion. The authors highlighted the importance of the food matrix consumed along with the NMs (i.e., food matrix effects) or their digestion products on the CNMs’ gastrointestinal fate and potential toxicity. The effect of the food matrix on NMs’ fate has already been stated by others [124,127,140,141]. In fact, this effect has been shown to mitigate cytotoxicity and also affect the digestion and absorption of food components and contaminants [134,142,143]. Particularly, multiple biological processes related to nutrient absorption and fate—such as fatty acid metabolism, glycolysis, protein transport, and protein catabolic processes—seem to be affected by the interaction between CNCs and the food matrix [134]. Using the same in vitro system and a high-fat food model, a reduction in triglyceride hydrolysis by CNFs and CNCs during the small intestinal phase was noted, which was more pronounced with smaller CNFs than with other CNFs or CNCs. This suggests a specific nanoscale effect, possibly related to the surface area per unit mass or the specific surface area (SSA) [10]. CNCs were found to modulate digestion viscosity and glucose concentration after in vitro digestion simulation. Smaller CNCs proved to be more effective in retarding glucose and diffusion, as demonstrated by their having the lowest starch hydrolysis rate and glucose diffusion rate [92]. CNMs were also shown to impact the GIT components, including bile salts, digestive enzymes, and mucins [124]. CNCs added to starch at a concentration of 0.28% prior to the starch gelatinisation process inhibited α-amylase and glucoamylase activities by 28% and 10–15%, respectively, reducing the digestibility of starch [144].

CNFs and CNCs could also sequester bile salts, as observed for some dietary fibres [10,135]. CNFs slightly reduced lipase activity at concentrations above 1.1% (*w*/*w*), showing a retardation effect on bile acid diffusion, measured as the ratio between the bile acid content (taurocholic acid) in dialysate in the presence and absence of CNFs [136]. CNFs, TEMPO–CNF, and CNCs were shown to exhibit mucoadhesion properties under simulated gastric and intestinal conditions, with viscosity synergism between CNMs and mucin, where the CNCs showed the greatest effect [137]. In vivo rodent studies regarding the effects of CNFs on starch digestion and glucose absorption demonstrated contradictory results, showing no significant differences in the blood sugar level or decreased lean body mass, decreased intestinal D-xylose absorption, and altered glucose homeostasis [9,145].

Collectively, these studies shed some light on the in vitro interactions of CNMs with either the digestion fluids or the food matrix following oral exposure, highlighting the relevance of characterising the CNMs along the GIT, since their properties may vary substantially [124,140]. Therefore, CNMs’ interactions with their surroundings—including with other biomolecules present in the biological milieu—are expected to influence CNMs’ toxicokinetics, including their biodistribution, possible translocation to secondary organs, accumulation, degradation, and clearance [124]. However, since no toxicokinetic studies are currently available, evidence on the in vivo capacity of CNMs to cross the GIT’s physiological barriers and reach secondary organs is still lacking.

## 4. Hazard Assessment of CNMs

Toxicological studies aim to generate data that identify hazards and contribute to predicting the health effects from exposure to a given substance, such as CNMs, allowing reduction in the risk of human exposure [23]. The current toxicity testing approaches for NMs are also recommended for CNMs [14]. The battery of in vitro testing to establish the genotoxic potential of NMs for use in food and feed should cover different genotoxic mechanisms [146,147,148]. According to the EFSA, in a regulatory context, these should include three specific endpoints (i.e., gene mutation, and structural and numerical chromosome aberrations) [61]. In addition, cellular uptake studies should be included in the event of negative genotoxicity results [61]. Other relevant endpoints for in vitro testing include cytotoxicity/cell viability, (pro-)inflammation status, induction of oxidative stress, and impairment of the integrity of the gastrointestinal barrier [61]. A follow-up in vivo study should be carried out when at least one of the in vitro tests indicates genotoxic effects, or if it is not appropriate to test the NMs in vitro, unless it can be demonstrated by other means that the positive in vitro findings are not relevant for the in vivo situation [61]. In vivo studies should also be considered if in vitro results indicate compromised epithelial barrier integrity, release of (pro-)inflammatory mediators, or effects on immune cells or immune response [61].

Until very recently, studies on the potential hazards of CNMs have focused mainly on the respiratory tract upon inhalation. Only a few studies have described the evaluation of their toxic effects in the GIT upon oral exposure. Despite this, the potential toxicity of CNMs upon oral exposure has been explored. An overview of these in vitro and in vivo studies is provided in the following sections.

### 4.1. In Vitro Toxicity of CNMs in GIT Cells

A summary of the in vitro studies that are relevant to oral exposure to CNMs is provided in Table 2. The cytotoxicity was evaluated in 26 studies of CNCs (11), CNFs (12), or both (3) (Table 2). The most frequently applied cellular model was the human colon carcinoma Caco-2 cell line, which can be differentiated into morphologically and functionally mature cells that resemble the enterocytes lining the small intestine [128]. Other cell lines included the human colon carcinoma HCT116 cells, the foetal colon cell line FHC, human hepatocellular carcinoma HepG2 cells, human hepatic HepaRG cells, murine Kupffer cells’ liver macrophages (KUP5), and mouse hepatoma cells (Hepa 1–6 cells).

#### 4.1.1. Cellulose Nanocrystals (CNCs)

The cellular viability of non-differentiated Caco-2 cells was evaluated using a resazurin-based method, after 24 and 48 h of incubation with two needle-shaped CNCs. These CNCs were obtained from a pretreated grape pomace extract by 64–65 percentage by weight (wt%) sulfuric acid hydrolysis for 30 min or 60 min, followed by an ultrasound treatment [149]. No effect was observed on the cellular metabolic activity after exposure to 50 µg/mL and 200 µg/mL of CNCs, irrespective of the duration of the sulfuric acid hydrolysis [149]. The effects of the duration of the sulfuric acid hydrolysis (30 min, 60 min, or 90 min) on the cytotoxicity of needle-like CNCs isolated from wheat bran cellulose were also investigated using Caco-2 cells, after exposure to a concentration range of 50–5000 μg/mL of CNCs for 24 h [150]. Cell viability was not significantly affected after exposure to three CNCs at concentrations up to 2000 μg/mL, but was decreased (<80% viability) after exposure to 5000 μg/mL of CNCs [150]. Additionally, no cytotoxicity was observed after 72 h exposure to 0–5 μg/mL of CNCs obtained from cotton linters, using either undifferentiated or differentiated Caco-2/TC7 cells [151]. Another study did not find evidence of CNC uptake by differentiated Caco-2 cells or cytotoxicity up to 10 mg/mL [152]. Moreover, there was no evidence that CNCs would penetrate the mucus gel layer using an in vitro mucus diffusion model layer, suggesting that mucus might be effective in blocking CNCs from further touching the intestinal cell lining [152].

Surface charge can affect CNMs’ colloidal stability under physiological conditions, which might impact on their biological effects [153]. Six different forms of CNCs were synthesised from softwood pulp with various amounts of surface carboxyl groups (1.7 to 6.6 mmol/g), and Caco-2 cells were exposed for 24 h to a wide range of concentrations (50–300 µg/mL) [153]. A charge-dependent decrease in mitochondrial activity was found for carboxyl contents higher than 3.8 mmol/g, suggesting that CNCs with surface carboxyl contents above a certain threshold may not be fully biocompatible, despite negligible effects on cell membrane integrity and low cell uptake of CNCs [153]. Moreover, the cytotoxicity of four CNCs with different sizes (108.4–1174 nm) was assessed in HCT116 colon adenocarcinoma cells. After 24 h of exposure, none of the CNCs presented cytotoxicity up to 250 µg/mL, but three were cytotoxic at 500 and 1000 µg/mL [154]. The cytotoxicity observed for the largest CNC was attributed to its tendency to gel formation at high concentrated suspensions, which may block the gas exchange through the cell membranes [154]. No cytotoxic effects were observed in HCT116, HT-29, and CCD112 colon fibroblast 2D cells after exposure to 7.8–500 µg/mL of CNCs obtained from rice straw waste, except for HT-29 cells at the highest dose. Similarly, CNC was not cytotoxic to 3D spheroid models of HCT116 and HT-29 cells for the same concentration range [155]. Furthermore, no cytotoxicity was observed in HCT116 colon cells after exposure to CNCs or CNCs grafted with poly(acrylic acid) up to 450 µg/mL, investigated for their potential application as mucoadhesive materials for the local delivery of cisplatin in colorectal cancer [156].

More complex intestinal models, such as tricultures of the small-intestinal epithelium (i.e., Caco-2, HT29-MTX, and Raji B cells), were also used to investigate CNCs’ cytotoxicity. For the exposure of this triculture, DeLoid et al. (2019) used a static in vitro simulated digestion process on CNCs (average diameter 25 nm) obtained from softwood bleached kraft fibre. The CNMs were added either to an FFM or an SFM, at 0.75 or 1.5% *w*/*w* concentrations. After 24 h of exposure, no differences were observed in the epithelial barrier integrity, and a very moderate increase in cytotoxicity and an increase in ROS production were observed using the FFM [62]. No increase in cytotoxicity or in ROS production was observed after 6 h of exposure when CNCs were dispersed in the SFM, suggesting that the food matrix had an impact on the biological consequences of these CNCs [62]. Likewise, another study strengthened the hypothesis that the food components in the SFM may ameliorate the adverse effects of CNCs using the same cell system, eliminating the slight cytotoxicity observed with CNCs in the FFM [134]. In the latter study, only the highest concentration of CNCs (312.5 μg/mL) in the FFM digesta induced a small but significant increase in cytotoxicity in the triculture after 24 h of exposure [134]. Moreover, although ingestion of CNCs did not cause significant perturbations of the cellular proteome in either food model, 125 proteins were significantly differentially expressed only in the culture exposed to CNCs, indicating significant interactions between CNCs and the food matrix—particularly in biological processes related to nutrient absorption and utilisation, such as protein transport, glycolysis, and fatty acid metabolism [134]. Using the same triculture model and the in vitro digestion process, no cytotoxicity was observed with CNC concentrations of 0.75% and 1.5% *w*/*w* [52]. Ede et al. (2020) also used the triculture cellular model of the epithelium described above, but a different static in vitro simulated digestion process [126]. No cytotoxicity was observed following exposure to CNCs for 1, 6, 24, or 48 h [138]. Additionally, no ROS formation was noted after different exposure timepoints (15 min, 30 min, 1 h, and 4 h). Barrier integrity, as measured over 7 days after exposure, was generally maintained. At day 2 post-exposure, a momentary decrease in transepithelial electrical resistance (TEER) was observed, attributed to the interference of exposure to CNCs with the electrical resistance measurement, and not reflective of changes in cell co-culture barrier integrity. The results showed minimal pro-inflammatory responses, with similar IL-6 expression to the negative and vehicle control treatments [138].

Other cellular systems than intestinal cell lines have been used less frequently to study CNMs’ cytotoxicity. Nanohybrid CNCs loaded with tannic acid had no cytotoxic effects in HepG2 cells up to 30 mg/mL [157]. Cell viability studies were undertaken in hepatocyte (Hepa 1–6) cell lines and transformed Kupffer cells (KUP5) following exposure to different-sized CNCs over a dose range of 0–200 µg/mL [158]. No cytotoxic effect was observed in Hepa 1–6 cells after exposure to CNCs, while in KUP5 cells decreased cell viability was observed for all CNMs with the ATP assay, but not with the MTS ((3-(4,5-dimethylthiazol-2-yl)-5-(3-carboxymethoxyphenyl)-2-(4-sulfophenyl)-2H tetrazolium) assay. Significant morphological alterations in KUP5 cells were induced by CNC samples, demonstrating cell shrinkage and convolution [158]. Cellular uptake of CNCs in KUP5 cells occurred mainly via phagocytosis and, to a lesser extent, clathrin-mediated endocytosis, as verified in Hepa 1–6 cells. In both cells, CNCs induced mtROS generation, caspase-3/7 activation, and apoptotic cell death. The phagocytosis of CNCs by KUP5 cells triggered lysosomal damage, cathepsin B release, caspase-1 activation, and IL-1β production [158].

Only one study was found addressing the mutagenicity of CNCs using the bacterial reverse-mutation test (OECD 471). CNCs did not show mutagenicity in strains of *Salmonella typhimurium* and *Escherichia coli* within the 0.13–5 mg/plate concentration range [159].

#### 4.1.2. Cellulose Nanofibres (CNFs)

Only 15 studies have addressed the cytotoxicity of CNFs in cells from the GIT. Different CNFs isolated from unripe banana peel were prepared for developing reinforced polymeric matrices (e.g., biodegradable films for food packaging), by either chemical or chemical and mechanical treatments, and their cytotoxicity was tested with undifferentiated Caco-2 cells exposed to 50–5000 µg/mL for 24 h [160]. Lower concentrations (50–1000 µg/mL) did not impact cell viability, but CNFs prepared with a lower sulfuric acid concentration (0.1%) significantly decreased cell viability (<70%) when applying concentrations above 2000 µg/mL, independently of whether or not a mechanical treatment was used. The less negatively charged CNFs, produced without mechanical treatment and using the highest acid concentration (10%), were not cytotoxic at any of the tested concentrations (50–5000 μg/mL), suggesting that higher acid concentrations and lower amounts of lignin and hemicellulose favoured cell proliferation [160]. The same group investigated the viability of Caco-2 cells after 24 h of exposure to two different CNFs isolated from unripe banana peel bran starch via enzymatic hydrolysis using xylanase. No cytotoxicity was observed at concentrations up to 2000 μg/mL, and only a slight significant decrease was observed at 5000 μg/mL [35]. No cytotoxicity was observed after exposing Caco-2 cells to 0.05–500 µg/mL of CNFs (3% *w*/*w*) produced from softwood kraft pulp for 48 h [145]. Another study evaluated the cytotoxicity of CNFs with different surface modifications, using the same cellular model [44]. CNFs were prepared from commercial never-dried bleached sulphite dissolved softwood pulp, using different methods [44]. Cellular metabolic activity was not significantly affected after 24 and 48 h exposure to a concentration range of 50–500 µg/mL of CNFs, except for the anionic CNFs produced by carboxymethylation, which displayed decreased metabolic activity (<70%) after 48 h of exposure to the highest concentration, although the cells preserved their normal morphology [44]. In contrast, no differences were observed in the viability of Caco-2 cells exposed for 24 h to four carboxymethylated CNFs with different carboxyl contents obtained from cotton linter pulp, at a concentration range of 100–1000 μg/mL [43]. Moreover, no toxicity was observed for CNM-based composites—particularly for CNFs/AgNPs or CNFs/TiO_2_—in either Caco-2 cells or FHC colon cells exposed to concentrations up to 1000 μg/mL, although one study described increased cytotoxicity at higher doses of CNFs/AgNPs [17,161,162].

DeLoid et al. used a static in vitro simulated digestion process on CNFs (average diameter = 64 nm) obtained from softwood bleached kraft fibre, to expose a triculture of Caco-2, HT29-MTX, and Raji B cells. The CNMs were added to either an FFM or an SFM, at 0.75 or 1.5% *w*/*w* concentrations, for 24 h. No differences were observed in the epithelial barrier integrity or ROS production with FFM digesta compared to controls [62]. Using the same triculture model and the in vitro digestion process, no cytotoxicity was observed with CNFs—either unlabelled or fluorescence-labelled—at concentrations up to 1.5% *w*/*w* in food [51], or with FITC-tagged CNFs at concentrations of 0.75% and 1.5% *w*/*w* [52]. Pradhan et al. (2020) also used the triculture cellular model of the epithelium described above, but with a different static in vitro simulated digestion process [126]. The group investigated the toxic effects of six industrially produced fibrillated cellulose materials ranging from 8 to 67 wt%, with no decrease in cell viability, barrier disruption, or inflammation observed following 1, 6, 24, or 48 h of exposure. Additionally, no oxidative stress was noted after different exposure timepoints (15 min, 30 min, 1 h, and 4 h) [163].

Using the hepatic HepG2 cells, no cytotoxic effect was observed after 24 h exposure to 0.01–0.5 wt% of TEMPO-oxidised CNFs extracted from sugarcane bagasse [164]. CNF hydrogel isolated from bleached birch pulp did not show cytotoxicity to HepaRG and HepG2 cells at concentrations of 0.1–1 wt% after 30 and 5 days of exposure, respectively [165]. In hepatocytes (Hepa 1–6 cells), no cytotoxic effect was observed with different-sized CNFs in a concentration range of 0–200 µg/mL, while in transformed Kupffer cells (KUP5 cells) decreased cell viability was observed for all tested CNMs with the ATP assay, but not with the MTS ((3-(4,5-dimethylthiazol-2-yl)-5-(3-carboxymethoxyphenyl)-2-(4-sulfophenyl)-2H tetrazolium) assay [158]. CNCs showed more significant toxicity in KUP5 cells than CNFs [158].

Lopes et al. evaluated the in vitro biological effects of unmodified and modified CNMs on the human gut bacteria *Escherichia coli* and *Lactobacillus reuteri*, by measuring bacterial growth and determining colony-forming unit counts after exposure to CNFs at 50 µg/mL and 500 µg/mL [44]. A bacteriostatic effect was observed for *Escherichia coli*, while no effect was observed for *Lactobacillus reuteri* [44]. In contrast, after in vitro exposure to a high concentration of a cellulose nanofibril/titanium dioxide NM nanocomposite (10 mg/mL) (for application in PVA-based films), no appreciable effect was observed on the growth of *Escherichia coli* P-24, *Lactobacillus acidophilus* ADH, or *Bifidobacterium animalis* Bif-6 [161]. More studies are needed to understand the impact of the CNMs, which might impact the microbiota in a beneficial or detrimental manner.

Three studies applied the bacterial reverse-mutation test (OECD 471) and one applied the mouse lymphoma TK assay (OECD 490) to assess the in vitro mutagenicity of CNFs, all with negative results [164,166,167]. CNFs produced via TEMPO oxidation and via mechanical defibrillation of needle-type bleached kraft pulp showed that CNFs at 3.13–100 μg/mL did not induce bacterial mutations when applying the bacterial reverse-mutation test (OECD 471) in multiple *Salmonella typhimurium* strains and *Escherichia coli*, nor did they induce mammalian mutations using the mouse lymphoma TK assay [166]. In accordance, no mutagenicity was observed using the Ames test following 48 h of exposure to 12.5–100 μg/plate of TEMPO-oxidised CNFs extracted from sugarcane bagasse, whether in the presence or absence of metabolic activation [164]. Another study applied the bacterial reverse-mutation test (OECD 471) in *Salmonella typhimurium* (TA102) with different fibrillated cellulose fractions obtained from mechanically treated bleached hardwood kraft pulp at several concentrations (19–300 µg/mL), showing no genotoxicity [167]. It should be noted that the Ames test is not considered suitable for NMs by the scientific community and the regulatory authorities to address NMs’ genotoxicity, owing to the inability of bacterial cells to internalise NMs [61,146,168,169].

Overall, nine studies have revealed cytotoxic effects, but most studies suggest that CNMs do not have cytotoxic effects in GIT cells, as defined by the ISO standard 10993-5, i.e., they do not reduce cell viability by more than 30%. Most positive findings are observed after exposure to high concentrations of CNMs (>2000 µg/mL) and in functionalised CNMs (e.g., CNCs with surface carboxyl contents above a certain threshold, or anionic CNFs produced by carboxymethylation). Studies on hepatic cells are scarce but suggestive of a possible toxic effect, which should be further investigated. No genotoxicity studies using GIT cells have been found. CNMs were not mutagenic in the bacterial Ames test or the mouse lymphoma TK assay (OECD 490).

Despite these initial negative findings, other regulatory relevant endpoints—such as DNA or chromosomal damage—were not assessed in any of the abovementioned studies. No significant or moderate ROS generation was observed in the three studies addressing oxidative stress. Additionally, no cell membrane integrity disruption or inflammation was observed, although these endpoints need further investigation. Most in vitro studies did not consider the effects of digestion on the physicochemical properties of ingested CNMs. Thus, CNMs’ toxicity remains unclear and merits further investigation to ascertain the potential adverse effects of innovative CNMs in the GIT.

### 4.2. In Vivo Toxicity of CNM

Several in vivo studies on the toxicity of CNMs have been reported following oral exposure (summarised in Table 3); 11 studied CNCs, while 10 studies were found for CNFs.

#### 4.2.1. Cellulose Nanocrystals (CNCs)

Two toxicity studies investigated the acute (OECD TG 425) and repeated oral toxicity (OECD TG 407) of CNCs that were produced via the sulphate process [159]. In the acute toxicity test, a single dose of 2000 mg/kg of CNCs in aqueous suspension was administered to rats by oral gavage, and the animals were monitored for 14 days. In the repeated oral toxicity test, daily doses of 500, 1000, and 2000 mg/kg of CNCs were administered by oral gavage for 28 days. No adverse effects were observed during rat maintenance (e.g., toxicity, altered neurological function, body weight, or food consumption) and after gross necropsy, whether after acute (LD50 greater than 2000 mg/kg bw/day) or repeated exposure (NOEL above 2000 mg/kg bw/day) [159]. The in vivo mammalian erythrocyte micronucleus test (OECD TG 474) was also applied by the same authors for the detection of chromosome damage induced by CNCs in the bone marrow and/or blood cells of rats, with negative results up to a maximum dose of 2000 mg/kg [159]. Acute oral toxicity testing (OECD TG 425) was also performed in mice exposed to several types of CNC produced from cotton microcrystalline cellulose, showing no changes in body weight or toxicity during maintenance of the mice [55]. No signs of toxicity were observed in the heart, spleen, or liver, although there was a significantly decreased mass coefficient of the kidneys in female mice receiving CNCs produced by solvolysis with the acetic acid/phosphotungstic acid system (disc-like cellulose-II allomorph D-CNCAc) [55]. No toxicity was observed in a subsequent subchronic oral toxicity study (OECD TG 408) conducted on rats exposed to 2, 3, and 4% CNCs through diet, for 90 days, with a calculated no-observed-adverse-effect level (NOAEL) of 2085.3 (males) and 2682.8 mg/kg bw/day (females) [138]. Ede et al. did not observe any histological alterations in the rats’ reproductive organs after oral exposure to CNCs for 90 days [138]. The target doses were selected on the basis of two range-finding studies (based on OECD TG 407), which determined no adverse effects associated with feeding 5% CNCs or up to 1.2% CNCs after 7 days and 14 days, respectively [138]. The heart, kidneys, and spleen were significantly heavier in the 4% CNCs group compared with the control group (feed with food-grade cellulose), despite no observable dose-related trend. Vacuolisation of periportal hepatocytes (variablysized, clear cytoplasmic vacuoles) was present in the livers of both 4% groups (CNCs and control), without the presence of hepatocyte degeneration or any other pathological observations [138]. No long-term studies of oral exposure to CNCs were found in the literature.

In a study by Zhang et al. [170], CNCs produced by sulfuric acid from bleached softwood kraft pulp and with a cationisation agent were administered by gavage to a murine model with chronic renal failure and hyperphosphatemia, every other day for 10 days, to evaluate their use as alternatives to phosphate binders for chronic renal failure and hyperphosphatemia therapy. The cationic CNCs treated hyperphosphatemia effectively without affecting the metabolism of trace elements, with no deleterious effects on the liver and intestines, and restored the levels of triglycerides in murine sera [170].

Potential in vivo hepatotoxicity of CNCs modified with oxalate esters was addressed by applying a 7-day repeated oral exposure in rats at doses of 50 and 100 mg/kg bw, and plasma and liver tissue samples were assayed using biochemical analysis, liver histopathology, and protein expression. No changes in the liver’s relative weight, alkaline phosphatase activity, and lipid peroxidation levels were observed at either dose level. However, at the highest dose, hepatic injury was observed with necrosis and severe cellular infiltration [59]. For the highest tested dose, CNCs modified with oxalate esters significantly elevated aspartate aminotransferase, alanine aminotransferase, and myeloperoxidase activities and enhanced the immunohistochemical expression of inducible nitric oxide synthase and Bcl-2-associated X protein in the liver [59]. The same group investigated the effects of CNCs from *Polyathia longifolia*, synthesised with sulfuric acid, in the cortex and cerebellum of albino rats after 14 days exposure to 50 and 100 mg/kg bw by oral gavage, observing elevated aspartate aminotransferase, cortical and cerebellar glutathione, and lipid peroxidation levels, but normal histology of the neurons, hippocampus, and Purkinje layers, with no alterations of body and organ weights, albumin, cortical and cerebellar catalase, and glutathione S-transferase levels [171].

Concerning nanocomposites, acute oral toxicity of lignin-coated CNCs was assessed applying the EPA’s test guideline OPPTS 870.1100—Acute Oral Toxicity, Up and Down Procedure—using female albino rats, orally administered with a single dose (5000 mg/kg) by oral gavage. There were no signs of gross toxicity, adverse clinical effects (including skin and eye irritation), abnormal behaviour, or alteration of organs of the thoracic and abdominal cavities after necropsy at the end of the 14-day observation period (acute oral LD50 > 5000 mg/kg) [172].

Currently, information on other health endpoints associated with chronic effects, carcinogenicity, neurotoxicity, and reproductive effects upon oral exposure is scarce.

Some studies evaluated the potential impacts of exposure to CNCs on the gut microbiota. Fermented CNCs were shown to significantly enhance the production of bacterial metabolites—such as short-chain fatty acids (including acetate, butyrate, and propionate)—and *Bifidobacterium* counts in faecal matter obtained from three healthy human donors, fermented under anaerobic conditions [173]. These short-chain fatty acids are recognised as important compounds that modulate the gut’s physiological functions, and are believed to be associated with multiple health effects related to gut barrier function, glucose homeostasis, immunomodulation, and obesity [173,174]. In vivo studies using male Wistar rats with oral administration of CNCs by gavage twice daily, applying a concentration of 250 mg/kg bw, significantly increased short-chain fatty acids in contrast to both controls and the microscale cellulose [173]. The effects of CNC intake on the gut microbiota were also assessed in high-fat-diet-fed mice treated with 0.1% or 0.2% CNC dispersions via drinking water for seven weeks, showing that administration of 0.2% CNCs—but not 0.1% CNCs—increased bacterial diversity and induced changes in the gut microbiota composition of high-fat-diet-fed mice, with decreases in the relative abundance of Streptococcaceae and Rikenellaceae and increases in Lactobacillaceae upon higher intake of CNCs. Moreover, there was a weight gain suppression in high-fat-diet-fed mice, treated with 0.2% CNCs compared to the untreated group, with a lower accumulation of epididymal and subcutaneous fat, suggesting that the consumption of CNCs had an inhibitory effect on obesity, which was mediated via regulation of the gut microbiota balance [175].

#### 4.2.2. Cellulose Nanofibres (CNFs)

Concerning CNFs, Andrade et al. (2015) found no evidence of toxicity in male mice fed with a diet containing 7%, 14%, or 21% CNFs derived from peach palm residue, based on biological, biochemical, and liver histological analyses performed after 30 days of exposure [9]. No adverse effects were observed in a study conducted according to the standardised OECD TG 408 in rats subchronically exposed to 2%, 3%, or 4% CNFs for 90 days, with mean dietary intakes of 1044, 1550, and 2194 mg/kg/day for males, respectively, and 1302, 1886, and 2667 mg/kg/day for females, respectively (NOAEL: 2194.2 mg/kg/day in males; 2666.6 mg/kg/day in females) [176]. In this study, no differences were found in survival, clinical observations, body weight, food consumption, ophthalmologic evaluations, haematology, serum chemistry, urinalysis, post-mortem anatomic pathology, or histopathology, including inflammatory or proliferative changes, compared to dietary administration of conventional forms of cellulose [176]. In male rats given 1% *w*/*w* CNFs by oral gavage, either in aqueous suspensions or as a heavy cream (as a high-fat food model), no relevant differences were observed in whole blood markers, serum markers (i.e., hepatic function, lipids, renal function, or electrolytes), or the histology of the liver, spleen, kidneys, and small intestine, although a moderate reduction in weight gain was observed for rats receiving CNFs compared to the rats receiving only water, only 20% fat, or 1 *w*/*w*% CNF concomitantly with 20% fat [62]. Interestingly, a study by the same authors showed that the exposure by single gavage to 1% CNFs synthesised by mechanical grinding reduced digestion of fat and blunted the postprandial rise of serum triglycerides in the same animal model, following the same high-fat diet (heavy cream), lowering fat absorption [10]. Subsequently, the same group demonstrated that exposure to CNFs leads to significant dysregulation in the expression of several genes involved in epithelial cell junctions, namely, several claudins, gap-junction proteins, and integrins, all involved in paracellular transport [122]. Moreover, CNFs caused sharp downregulation of one of the major cadherins (Cdh1) involved in the maintenance of tight junctions, and of the adherent junction protein Nectin-2 (Pvrl2) [122]. Increased cytokine production, modulating the proliferation of CD8+ T cells, was also observed [122].

Four carboxymethylated CNFs obtained from cotton linter pulp with different carboxyl contents (0, 0.36, 0.72, and 1.24 mmol/g) were administered to female mice by oral gavage once per day for eight weeks, in the form of 1% or 3.5% *w*/*w* CNFs suspensions in water, with no alterations in haematology or serum markers when compared to controls [43]. In another study, mice exposed to a subchronic treatment (4–6 weeks) of 30 mg/kg bw/day of CNFs by oral gavage showed some adverse effects, such as dysregulated glucose homeostasis and decreased lean body mass—particularly in male mice—although no differences in food consumption were observed [145].

Some studies addressed the effects of CNFs on colon inflammation in inflammatory bowel disease models. C57BL/6 mice in a colitis model (induced with dextran sulphate sodium, DSS) were administered with 0.1% CNFs in the water, obtained from adlay, seaweed, pear, or wood, for 5 days [177,178]. Compared to DSS-treated animals, administration of CNFs suppressed colon damage and exerted anti-inflammatory effects via suppression of NF-κB activation, except when the CNFs were obtained from wood [177,178]. Exposure to these CNFs additionally suppressed the myeloperoxidase activation of inflammatory cells, such as leukocytes.

Concerning nanocomposites, the acute oral toxicity of lignin-coated CNFs was assessed by applying the EPA’s test guideline OPPTS 870.1100—Acute Oral Toxicity, Up and Down Procedure—as described above for CNCs, with no adverse effects reported [172].

Currently, information on other health endpoints associated with chronic effects, carcinogenicity, neurotoxicity, and reproductive effects upon oral exposure is missing for CNFs, to the best of our knowledge.

One study addressed the potential impact of exposure to CNFs on the gut microbiota. Ingested CNFs caused noticeable changes in genus and species diversity, as well as in the abundance of specific notable bacterial species and genera. In rats that received 1% (*w*/*w*) CNFs alone or concomitantly with cream, the populations of *Coprococcus catus* and *Bacteroides acidifaciens*—which normally play a protective role in the GIT—were sharply reduced compared to rats exposed to gavage with water [122].

As can be deduced from the majority of the described studies, the subacute (<28 days) and subchronic (<90 days) oral toxicity of different forms of unmodified CNMs or carboxymethylated CNFs did not reveal major adverse effects, even when included in a relatively high percentage of the diet. Studies on carcinogenicity with long-term exposure (>90 days) and more realistic low-dose concentrations are still lacking. Other endpoints—such as genotoxicity, neurotoxicity, and reproductive effects—have also not been extensively addressed, and constitute knowledge gaps in hazard identification that hamper adequate risk assessment of CNMs. Additionally, few studies have considered the impact of exposure to CNMs in pre-existing intestinal diseases, such as Crohn’s disease and ulcerative colitis. The performed in vivo studies indicate an overall lack of toxicity upon oral exposure to CNCs or CNFs. However, exposure to CNFs was shown to negatively impact the expression of several genes involved in epithelial cell junctions and inflammation. Additionally, hepatotoxic effects were also observed by one group at a high dose (100 mg/kg bw) of functionalised CNCs after a 7-day repeated oral exposure. These findings merit further investigation.

## 5. Relevant Features to Be Considered in the Toxicity Assessment of CNMs

In view of the dynamic specific morphological and chemical characteristics of most NMs, which are often context-dependent, particular attention should be given to several issues that may alter their biokinetic behaviour and/or toxicological responses and may bias CNMs toxicity studies. These are summarised in Table 4.

As in any chemical toxicology evaluation, it is important to guarantee that CNM samples are not contaminated with chemical or biological substances that could impact their toxic behaviour in the test systems. Storage conditions, the absence of antimicrobial agents, and biological contaminants such as endotoxins should be checked [14,22]. The levels of impurities should also be carefully controlled [22]. Among the 40 revised studies, 32 did not indicate sample purity or addressed chemical or biological contaminations, while 6 studies investigated the endotoxin levels using the EndoZyme^®^ recombinant factor C assay [10,51,52,62,122,134], 1 assessed the endotoxin levels using the limulus amebocyte lysate (LAL) [158], and 8 performed microbiological analysis [10,51,52,62,122,134,138,176]. In addition, five studies used methods such as filtration, ultraviolet light, or autoclaving for sterilising the CNM samples [43,44,55,138,164,165], and only one applied a biocidal product [167].

In general, CNMs are produced in an aqueous suspension form, with common concentrations ranging from 0.5 to 3 wt% for CNFs and from 1 to 2 wt% for CNCs [14]. CNCs can also be stocked as a dispersible freeze/spray-dried powder, while CNFs with concentrations ranging from 1 to 25 wt% are normally presented as a gel or paste [14]. Independently of the CNMs’ initial state (i.e., wet or dry), a prerequisite for toxicological studies is an effective and reproducible dispersion method using physiologically relevant conditions, either in the dispersant vehicle of choice or in a biological medium [179]. Sample preparation and dispersion have been considered relevant issues in nanotoxicology for many years [128,180]. This is currently a challenge for CNMs as well, due to their physicochemical properties and the appropriateness of the methods to assess their dispersion, either in biological media or when CNMs are submitted to in vitro models of processes such as digestion [14,127,179]. Different methods have been used to disperse CNMs prior to toxicological studies, such as magnetic stirring [181], vortexing [179], or ultrasonication [57,58,149,179,182]. The latter has been the gold standard method for the dispersion of metal and metal oxide NMs and carbon-based materials, such as carbon nanotubes (CNTs), based on harmonised procedures such as the Nanogenotox dispersion procedure [183]. However, the applicability of ultrasonication for the dispersion of CNMs has been questioned, particularly for CNFs [179]. Ultrasonication (10 min) and subsequent high-speed vortexing led to webs of CNF entanglements spanning several μm, along with increased agglomeration of CNCs [179]. Instead, high-speed vortexing of CNFs for 20 s in deionised water (concentration 0.1 mg/mL) or 60 s in medium was preferred, whereas 600 s or 120 s of vortexing led to fibril entanglement and increased particle size distribution [179]. In turn, CNCs were adequately dispersed by “tube inversion”, and dispersion in cell culture medium only required 10 s of high-speed vortexing to evenly disperse the particles, as verified by macroscopic observation [179]. Whether the use of these methods for toxicological studies can be extrapolated to other CNMs is still uncertain, considering the enormous diversity of CNMs being produced. Thus, a standardised procedure for low concentrations of CNMs—particularly in biological media—is still lacking, and should be further investigated [14,27,179]. Nevertheless, the dispersion method used should be properly reported to allow comparison of data from different studies. In the studies reviewed here, 11 reported the methods used, while the majority (29 studies) did not, hindering the comparison of the results obtained.

The physicochemical characteristics of NMs are of utmost importance for safety assessment, since it is well-recognised that these can strongly influence their biological impact—particularly their toxicological properties [14,24,184,185]. Moreover, understanding which physicochemical characteristic(s) could potentially contribute to NMs’ adverse effects could assist in designing out specific properties in the development of safer NMs for commercialisation. We have previously shown that NMs with the same chemistry but with different primary properties may yield different biological effects [184,186,187,188]. Additionally, the characterisation of NMs under the conditions that are physiologically relevant to the exposure scenario during a toxicological evaluation is relevant to understand their interactions with living systems [179].

The most relevant aspects that can affect the toxicity of CNMs are their dimensions and morphology (i.e., width, length, aspect ratio), colloidal stability in media, surface chemistry, specific surface area, and degree of crystallinity (directly related to the material’s stiffness) [14,25,27,123]. CNMs’ size—as given by width, length, and aspect ratio—is thought to affect their potential hazards [14]. CNFs’ thickness and length apparently modulate their interactions with dendritic cells [189], while the largest (1174 ± 338.7 nm) and smallest CNCs (108.4 ± 94.8 nm) were considerably more cytotoxic than the medium-sized samples at concentrations of 500 and 1000 μg/mL [154]. Different lengths of CNMs showed different interactions with Kupffer cells and hepatocytes, with CNCs triggering cytotoxicity while CNFs failed to induce significant cytotoxicity due to their minimal cellular uptake [158].

CNMs’ surface chemistry influences their colloidal stability, rheology, and interfacial properties. The surface charge depends on the type of functional group present in the CNMs, which is determined by their interactions with the surrounding milieu. These functional groups are dependent on whether CNCs are produced by acid hydrolysis or oxidation, and the other used reagents, or on whether CNFs are TEMPO-oxidised, carboxymethylated, or have residual charge groups from hemicelluloses, among others [14,34]. For example, a charge-dependent decrease in mitochondrial activity was observed for -COOH contents higher than 3.8 mmol/g (∼50% reduction for -COOH contents of 6.6 mmol/g), although with a negligible effect on cell membrane integrity in Caco-2 cells [153]. Lopes et al. (2020) studied the effects of different surface modifications (i.e., carboxymethylation, hydroxypropyltrimethylammonium substitution, phosphorylation, and sulphoethylation) and found that cytotoxicity was only observed for carboxymethylated CNFs in Caco-2 cells [44].

The immunogenic potential of several functionalised CNFs and CNCs has been tested in vitro to elucidate whether and how their surface chemistry can influence their biocompatibility. Through surface modifications, CNCs’ internalisation by macrophages and their pro-inflammatory effects can be modulated [190].

Certain treatments prior to fibrillation of CNFs enhance the charges on the fibril surface, such as carboxymethylation and TEMPO oxidation, which can influence their biological effects. When carboxyl groups were introduced on the surface of CNFs, the tolerogenic potential of dendritic cells was shifted towards the induction of regulatory CD8+ T cells, whereas the introduction of phosphonates on CNFs’ surface potentiated dendritic cells’ capacity to induce both regulatory CD8+ T cells and type 1 regulatory (Tr-1) cells [190].

The source of the raw material can apparently influence CNMs’ toxicity, since they present different aspect ratios, surface functional groups, and charges, as determined by the extraction and modification methods. The investigation of the impact of different sources of CNCs (i.e., hemp, flax, and cellulose powder) showed that although CNCs did not cause cytotoxicity, flax CNCs exerted greater cell growth inhibition [191]. In contrast, Harper et al. found that the CNC source (i.e., cotton, wood pulp, or kraft pulp) had little impact on the toxicity to zebrafish [192]. The toxicological studies of CNMs reviewed here used a variety of sources, such as grape pomace [149], banana peel bran [35,160], wood pulp [51,138,163,176], hardwood birch pulp [165], softwood pulp [10,44,52,62,122,134,153], flax fibres [193], peach palm [9], cotton [43,55,59], sugarcane bagasse [164], and wheat bran [150]. Consequently, many different sizes, morphologies, and charges have been reported. As such, interpretation of data from toxicological studies with CNMs from different sources should be addressed carefully and should be accompanied by adequate information on the source, extraction methods, and the physicochemical characterisation of CNMs, as well as the dispersion methods used, as described above.

An adequate characterisation of CNMs’ properties is essential for establishing their physicochemical identity, both as pristine materials and when applied in complex matrices, such as product formulations. Thus, the identification of changes in the materials or products during storage, when used in in vitro or in vivo testing, and after ingestion is fundamental [61]. Moreover, as mentioned above, physicochemical features can affect biological interactions [61]. In the literature, there are reviews assessing the suitability of different techniques for determining the physicochemical characteristics of CNMs, along with their advantages, limitations, and common pitfalls [14,194]. The current techniques available for measuring particle size distribution and dispersibility, such as light-scattering- and particle-mobility-based methods for hydrodynamic size measurements, e.g., dynamic light scattering (DLS), are not optimal for measuring materials with anisotropic shapes, including flexible, soft, and fibrous materials such as CNMs [14,179,194]. Microscopic techniques are preferred for analysing CNMs’ morphology, providing information on their size and shape. Optical microscopy can be useful for a general perspective of their aspect, morphology, and size, as well as to evaluate the sample size/dimensional homogeneity [23]. However, suitable confirmatory methods are based on electron microscopy, such as scanning electron microscopy (SEM), transmission electron microscopy (TEM), and atomic force microscopy (AFM). TEM imaging can also be used to evaluate the aggregation state of CNC suspensions, clustering CNC aggregates into different groups according to their maximum Feret diameter (MFD), elongation, circularity, and area [47,194,195]. Nevertheless, in the case of CNFs, the determination of length using microscopic techniques is often difficult due to their entanglement and micrometre-scale; therefore, only the fibril width of CNFs is generally provided in the literature [23,194,195].

NMs’ surfaces interact with various ligands (e.g., natural organic matter, other NMs, proteins, etc.), which can affect their kinetics in media, their stability, their uptake into biological organisms, etc. [14]. Therefore, the surface charge should also be reported in toxicological studies of NMs. Conductometric titrations can be used to determine the surface charge density for sulphate half-ester groups or carboxyl groups, while the zeta potential is used for other functional groups [14]. Nevertheless, due to the high aspect ratio of CNMs and their sometimes-high surface charge density, zeta potential should not be considered as a quantitative measure of surface potential or surface charge density, but only as a relative assessment of colloidal stability [14]. Moreover, the possibility of adsorption of proteins to the NMs’ surface, forming coronas from biomolecules, is widely recognised as influencing the bioreactivity of NMs and, thus, as a relevant property for their characterisation, to better evaluate NMs’ biological outcomes [196]. NMs with similar physicochemical properties share relatively higher similarity in their protein corona compositions, as seen with CNCs and CNFs [196].

Dosimetric considerations such as dose range, rate, and the rationale commonly used to select a dose range are not always justified in the reported studies. When applied in food technology, such ranges should be justified or accounted for based on human daily dietary intake data for that specific NM [128]. Moreover, the utilisation of 3D cellular models instead of 2D cellular models may contribute to different toxicity outcomes, in view of the increased complexity of the systems used [197].

## 6. Concluding Remarks

CNMs are promising nanomaterials with a wide range of applications in multiple industrial sectors—particularly in food/food packaging and biomedicine. However, concerns related to their nanoscale properties have been raised. From this literature review, it is evident that the safety of ingested CNMs is still an open question, due to the lack of physiologically relevant toxicological data, and the effects of CNMs in the GIT remain unclear. Despite the increase in the research in this field in recent years, the number of available studies is limited, particularly in view of the variety of CNMs being developed.

In general, the existing in vitro and in vivo studies on the toxicity of ingested CNMs suggest negligible toxicological effects, favouring the biocompatibility of the majority of the tested CNMs. However, positive findings were reported, particularly related to high doses or to functionalisation of CNMs. Exposure to CNFs was shown to negatively impact the expression of several genes involved in epithelial cell junctions. Additionally, hepatotoxic effects were observed. Moreover, their genotoxicity has not been addressed and, as such, cannot be ruled out. Knowledge of other potential adverse health effects—such as inflammation or reproductive and carcinogenic effects—is very limited or still lacking. These merit further investigation. Moreover, there are no data from oral toxicity studies, particularly chronic feeding studies designed to assess long-term effects, and/or toxicokinetic (ADME) studies that would support the absence of toxic effects of ingested CNCs and CNFs.

Overall, the toxicity of CNMs appears to depend largely on their physicochemical properties which, in turn, depend strongly on the cellulose source, preparation procedure, or sterilisation, which need to be carefully controlled. Although most of the reviewed studies provide information on a minimum set of physicochemical characteristics of CNMs, so far, it is still unclear which might contribute more significantly to the biological interactions of CNMs within the GIT and their potential toxicity. Therefore, considering the differences in the manufacturing, purity, and physicochemical properties—particularly the surface chemistry of CNMs, as well as their secondary properties in biological media—it is recommended that they should be individually tested.

The inclusion of in vitro digestion simulation in the safety evaluation of ingested CNMs can be considered a valuable tool and an innovative approach to better understand the impact of the digestive process on the toxicity of CNMs and better reflect the modifications that CNMs may undergo in organisms, enabling a significant improvement of existing experimental approaches [132]. Furthermore, the use of advanced multidimensional cellular models—such as co-cultures and 3D cell cultures, with improved resemblance of in vivo GIT conditions [198]—can contribute to reducing inaccuracies in the hazard assessment of ingested CNMs for human health.

In this review paper, we provided a general overview on the current existing toxicological information on ingested CNMs, and identified the need for future investigations, using both in vitro and in vivo approaches, to comprehensively characterise their potential toxicity. Moreover, we addressed some of the key aspects that should be considered for the safety assessment of these materials, in the hopes of contributing to the scientific basis for future investigations of CNMs, supporting their sustainable and responsible application in the food and biomedical industries.

## Figures and Tables

**Table 1:** Interactions of CNMs with food digestion, and possible effects on CNMs’ properties and food components.

Effects	Description	Reference
Influence of the food matrix on the physicochemical properties of CNMs		
Interaction of the food matrix with CNMs	Larger particle agglomerates were observed in the food matrix when in the presence of CNCs, suggesting possible binding of CNCs to the nutrient particles (e.g., fat droplets)	[134]
Influence of CNMs on the digestion of food components		
CNMs’ interactions with fat	Reduction in triglyceride hydrolysis by CNFs and CNCs. CNMs can interact with fatty foods, thereby substantially reducing the digestion and absorption of fat	[10]
Impact of CNCs on lipid digestion	CNCs sequester bile salt and bind with protein-coated lipid droplets via bridging effects, restricting the available surface area for lipase	[135]
Influence of CNCs on the digesta’s viscosity and the subsequent release and diffusion of glucose	CNCs modulate the viscosity of the digesta. The release and diffusion rates of glucose were significantly reduced in the CNC–food system, and the digestion and diffusion of starch and glucose were delayed.	[92]
CNFs’ effects on lipid digestion and absorption and related mechanisms	CNFs slightly reduced lipase activity and increased intestinal digesta viscosity, and had a bile-acid-retardation effect; CNFs led to higher cholesterol adsorption compared to cellulose, but did not affect cholesterol micellar solubility; CNFs did not affect the total amounts of free fatty acids produced during lipid digestion	[136]
Influence of digestion on the physicochemical and biological properties of CNMs		
Effect of digestion conditions on the mucoadhesion of CNMs	CNMs have mucoadhesive properties in the digestive tract, with the level of adhesion depending on the type and concentration of CNMs, as well as the gastrointestinal compartment; CNCs showed the highest viscosity synergism in the stomach, while TEMPO–CNFs displayed synergism only under gelling concentrations	[137]
Effects of in vitro digestion on CNMs’ size and surface charge	No effect	[138]
Effects of digestion on CNMs’ size and viscosity	No observable changes in the particle size of CNCs, CNFs, and CNF–TEMPO in all digestive compartments; In the stomach, CNF–TEMPO aggregated and formed phase separation, resulting in decreased viscosity and increased particle size; CNCs formed hydrogel networks, causing increased viscosity	[139]

**Table 2:** In vitro toxicity assessment of CNMs in GIT cellular models.

CNMs	Endpoint (Assay)	Cellular Model	Source; Isolation Method; Isolation Conditions	Characteristics	Dispersion Method	Concentrations Tested	Endotoxin and Sterility Check	In Vitro Digestion	Exposure Duration (h)	Main Results	Reference
CNCs											
CNC1 CNC2 CNC3 CNC4	Cytotoxicity (WST-1)	HCT116	Fluka Avicel PH-101; AH (CNC1: H_2_SO_4_; CNC2 H_2_SO_4_/HCl; CNC3:NAOH/H_2_SO_4_/HCl;CNC4: HC)	CNC1 *L*: 256 ± 64.8 nm; CNC2 *L*: 140.5 ± 37.5 nm; CNC3 *L*: 108.4 ± 94.8 nm; CNC4 *L*: 1174 ± 338.7 nm;	NA	10–1000 μg/mL	NA	No	24 h	Cytotoxic effects at concentrations equal to or above 500 μg/mL	[154]
CNCs with variable COOH contents	Cytotoxicity (LDH release; MTS; live/dead staining), cell uptake	Caco-2	Softwood pulp; AH (HCl)	*d*: 125 nm to 234 nm; *L*: 97 nm to 110 nm; *w*: 1 nm to 8 nm; –COOH content: 1.7 to 6.6 mmol/g	NA	100–300 μg/mL	NA	No	24 h	Charge-dependent decrease in mitochondrial activity for -COOH contents higher than 3.8 mmol/g; no cellular membrane disruption and low cell uptake of CNCs	[153]
CNC-AH30S CNC-AH60S	Cytotoxicity (resazurin)	Caco-2	Dry grape pomace residue; AH (H_2_SO4)	CNC-AH30S: *L*: 307 nm; *w*: 8 nm; *L*/*d*: 38; CI: 70.62% CNC-AH60S: *L*: 323 nm; *w*: 7 nm; *L*/*d*: 46; CI: 74.89%	Magnetic stirring followed by ultrasonication (15 min; 37 KHz; 104 W)	50–200 μg/mL	NA	No	48 h	No effects	[149]
CNC30 CNC60 CNC90	Cytotoxicity (MTT)	Caco-2	Wheat bran; AH (H_2_SO_4_)	CNC30: *L*: 644.77 ± 225.2 nm; *w*: 33.80 ± 9.83 nm; *L*/*d*: *20.39* ± 8.4; ZP: −36.5 ± 0.8 mV; Y: 37.11 ± 1.43% CNC60: *L*: 568.81 ± 229.66 nm; *w*: 21.57 ± 9.71 nm; *L*/*d*: *30.01* ± 13.88; ZP: −39.8 ± 1 mV; Y: 35.11 ± 0.95% CNC90: *L*: 486.18 ± 177.36 nm; *w*: 16.94 ± 7.30 nm; *L*/*d*: *32.11* ± 13.19; ZP: −39.6 ± 1.2 mV; Y: 28.7 ± 1.54%	NA	50–5000 μg/mL	NA	No	24 h	Cytotoxicity at 5000 μg/mL	[150]
CNC-25	Cytotoxicity (LDH release), oxidative stress (flow cytometry with CellROX^®^ green reagent) TEER	Caco-2/HT29MTX/Raji B	Softwood bleached kraft fibre; AH (H_2_SO_4_)	*L*: 267 ± 91 nm; *d*: 25.2 ± 9 nm; *L*/*d*: 11.5 ± 3.2; SSA: 93 m^2^/g	NA	0,75% and 1.5% (*w*/*w*)	Endotoxin levels using the EndoZyme^®^ recombinant factor C (rFC) assay; microbiological assessment	Yes	24 h	Moderate cytotoxicity increase and significant ROS increase at the highest dose in the fasting food model (<1.1-fold)	[62]
FITC-CNC	Cytotoxicity (LDH release) TEER	Caco-2/HT29MTX/Raji B	Softwood bleached kraft fibre; AH (H_2_SO_4_)	*L*: 267 ± 91 nm; *d*: 25.2 ± 9 nm; *L*/*d*: 11.5 ± 3.2; SSA: 93 m^2^/g	Homogenisation (vortex, 20 s)	0.75% *w*/*w* (7500 μg/mL) and 1.5% *w*/*w* (15,000 μg/mL)	Endotoxin level using the EndoZyme^®^ recombinant factor C (rFC) assay; microbiological assessment	Yes	24 h	No cytotoxic effects; epithelial integrity maintained	[52]
CNCs	Cytotoxicity (LDH release), TEER, oxidative stress (flow cytometry with CellROX^®^ green reagent), proteomics (liquid chromatography with mass spectrometry)	Caco-2/HT29MTX/Raji B	Softwood bleached kraft fibre; AH (H_2_SO_4_)	*L*: 267 ± 91 nm; *d*: 25.2 ± 9 nm; *L*/*d*: 11.5 ± 3.2; SSA: 93 m^2^/g	NA	156.25; 312.5 μg/mL	Endotoxin levels using the EndoZyme^®^ recombinant factor C (rFC) assay; microbiological assessment	Yes	24 h	Cytotoxicity and ROS induction at the highest dose in the fasting food model (<1.1-fold); 125 significantly differentially expressed proteins; epithelial integrity maintained	[134]
CNCs	Cytotoxicity (MTS), oxidative stress (flow cytometry with CellROX^®^ green reagent), TEER, inflammation (IL-6 expression)	Caco-2/HT29MTX/Raji B	Wood pulp; AH (H_2_SO_4_)	*L*: 25 to 250 nm; *w*: <10 nm; *w*: 893 ± 251 nm; PDI: 0.51 ± 0.02; ZP: −50.8 ± 6 mV, rod-shaped	Homogenisation (Vortex-Genie 2, 10 min)	0.02% (*w*/*w*)	Assessment of impurities and microbiological contaminants	Yes + lysosomal digestion	1 h, 6 h, 24 h, or 48 h	No effects in any of the endpoints analysed	[138]
CNCs-1 CNCs-2 CNCs-3	Cytotoxicity (ATP, MTT; activations of caspases), oxidative stress (H_2_DCFA; glutathione; mtROS), lysosomal damage, release of IL-1β and TNF-α	Hepa 1–6 cells and Kupffer cells (KUP5)	NA	CNCs-1: *L*: 149.0 ± 50 nm; *w*: 16 ± 5.0 nm; *L*/*d*: 9.3 ± 1.9; *d*: 121.9 ± 19.9; ZP: −14.3 ± 1.6 mV; CNCs-2: *L*: 279.1 ± 116.3 nm; *w*: 22.4 ± 7.2 nm; *L*/*d*: 12.7 ± 2.2; *d*: 249.5 ± 44.8; ZP: −12.8 ± 1.5 mV; CNCs-3: *L*: 715.0 ± 315.0 nm; *w*: 27.0 ± 8.0 nm; *L*/*d*: 26.5 ± 5.9; *d*: 780.2 ± 57.7; ZP: −11.7 ± 1.2 mV;	Vortexing + bath ultrasonication (15 min, 42 Khz, 100 W)	25–200 µg/mL	Endotoxin levels using the limulus amebocyte lysate (LAL)	NO	24 h	No cytotoxic effects in Hepa 1–6 cells; decreased cell viability and significant morphological alteration in KUP5 cells; mtROS induction, caspase-3/7 activation, apoptotic cell death, lysosomal damage, cathepsin B release, NLRP3 inflammasome and caspase-1 activation, leading to IL-1β production in KUP5 cells, cellular uptake, mtROS generation, and caspase-3/7-mediated apoptotic cell death in Hepa 1–6 cells	[158]
CNCs	Cytotoxicity (MTT)	Caco-2/TC7 (undifferentiated or differentiated)	Microcrystalline cellulose (MCC) from cotton linters; AH (H_2_SO_4_)	CNC type I: *L*: 200−300 nm; *w*: 5−10 nm; ZP: >20 mV CNC type II: *L*: 20−100 nm; *w*: 15−20 nm; ZP: >20 mV	NA	0–5 μg/mL	NA	No	72 h	No cytotoxic effects	[151]
CNCs	Cytotoxicity (MTT), cellular permeation	Caco-2 differentiated	Wood pulp; AH (H_2_SO_4_)	*L*: 150−200 nm; *w*: 5−20 nm; *d*: 98.9 ± 2.5 nm.	NA	1–10,000 μg/mL	NA	Yes, but not for biological studies	24 h	No cytotoxicity and no cellular permeation	[152]
CNCs	Cytotoxicity (clonogenic assay)	HCT116 HT-29 CCD112 colon fibroblasts	Rice straw waste; AH (H_2_SO_4_)	*d*: 109.64 ± 2.8 nm; ZP: −42.76 ± 1.4 mV	NA	7.8–500 µg/mL	NA	No	72 h	No cytotoxicity, except in HT-29 cells at the highest dose	[155]
CNCs CNCs grafted with poly(acrylic acid)	Cytotoxicity (MTT)	HCT116	Bleached wood pulp; AH (H_2_SO_4_)	*L*: 100–200 nm; *w*: 5–15 nm; density: 1.6 g/cm^3^, CI: >80% SSA: 200–300 m^2^/g; ZP: −28.37 ± 0.65 mV	NA	7.03–450 µg/mL	NA	No	72 h	No cytotoxic effects	[156]
CNCs/tannic acid	Cytotoxicity (MTT)	HepG2	Microcrystalline cellulose Avicel^®^ PH-101; AH (H_2_SO_4_)	*L*: 220 ± 67 nm; *d*: 156.72 ± 57.47 nm; ZP: 41.62 ± 0.42 mV	NA	10–30,000 µg/mL	NA	No	24 h	No cytotoxic effects	[157]
CNFs											
CNF	Cytotoxicity (resazurin; alamarBlue)	HepaRG HepG2	Bleached birch pulp; controlled homogenisation process using an industrial fluidiser	*w*: 20–30 nm	NA	0.1–1% (*w*/*w*)	Sterilised by autoclaving (121 °C, 20 min).	No	30 and 5 days	No cytotoxic effects	[165]
CNFs-N 0.1%; N 1%; N 10% CNFs-NM 0.1%; NM 1%; NM 10%	Cytotoxicity (MTT)	Caco-2	Unripe banana peel bran; AH (H_2_SO_4_ and/or MT: HPH)	*d*: 2.89 nm to 4.65 nm; *L*: 310.77 nm to 619.57 nm; *L*/*d*: 93.95 to 143.51; z-potential: 37.60 mV to 67.37 mV; Y: 27.07 to 71.51	NA	50–5000 μg/mL	NA	No	24 h	Cytotoxicity above 2000 mg/mL	[160]
CNF1 15% CNF2 35%	Cytotoxicity (MTT)	Caco-2	Banana peel bran with different concentrations (15% and 35%); enzymatic hydrolysis	CNF1: *L*: 1490.0 ± 107.3 nm; *d*: 3.7 ± 0.4 nm; *L*/*d*: 404 ± 63.9; ZP: −29.1.5 ± 0.7 mV; CI 61.5 ± 1.1 CNF2: *L*: 1544.5 ± 40.6 nm; *d*: 8.8 ± 0.7 nm; *L*/*d*: 170.2 ± 14.7; ZP: −31.5 ± 2.9 mV; CI: 66.2.5 ± 4.1	NA	50–5000 μg/mL	NA	No	24 h	Cytotoxicity at 5000 μg/mL (74.59% and 73.13%)	[35]
CNFs	Cytotoxicity (LDH release), oxidative stress (flow cytometry with CellROX^®^ green reagent), TEER	Caco-2/HT29MTX/Raji B	CNFs: softwood bleached kraft fibre; mechanical ultrafine friction grinding; autoclaved	*L*: 6710 ± 5611 nm; *d*: 64 ± 29 nm; *NtN*: 335.60 ± 232.66 nm; *L*/*d*: 107.6 ± 54.5; SSA: 34 m^2^/g	NA	0,75% and 1.5% (*w*/*w*)	Endotoxin level using the EndoZyme^®^ recombinant factor C (rFC) assay; microbiological assessment	Yes	24 h	No effects	[62]
CNF/Ag	Cytotoxicity (MTT; WST-8)	Caco-2 FHC	CNF slurry	*L*: 95.22 ± 0.29 nm; ZP: −21.13 mV	NA	50–1000 μg/mL	NA	No	24 h	No cytotoxic effects	[17]
FITC-CNF	Cytotoxicity (LDH release), TEER	Caco-2/HT29MTX/Raji B	CNFs: softwood bleached kraft fibre; mechanical ultrafine friction grinding; autoclaved.	*L*: 6710 ± 5611 nm; *d*: 64 ± 29 nm; *NtN*: 335.60 ± 232.66 nm; *L*/*d*: 107.6 ± 54.5; SSA: 34 m^2^/g	Homogenisation (vortex, 20 s)	0.75% *w*/*w* (7500 μg/mL) and 1.5% *w*/*w* (15,000 μg/mL)	Endotoxin level using the EndoZyme^®^ recombinant factor C (rFC) assay; microbiological assessment	Yes	24 h	No cytotoxic effects; epithelial integrity maintained	[52]
CNF–U enzymatic pretreatment CNF–A carboxymethylated CNF–C cationic CNF–P phosphorylated CNF–S sulphoethylated	Cytotoxicity (resazurin live/dead staining), effects on the human gut bacteria *Escherichia coli* and *Lactobacillus reuteri*	Caco-2	Never-dried bleached sulphite dissolved softwood pulp; enzymatic pretreatment; carboxymethylation, phosphorylation, sulphoethylation	CNF–U: *w*: 10–30 nm; ZP: −8.7 ± 1.3; CI: 61%; content functional groups: 30 mol/g CNF–A: *w*: 4–5 nm; ZP: −13.0 ± 0.8; CI: 52%; content functional groups: 570 mol/g CNF–C: *w*: 4–5 nm ZP: −11.7 ± 0.9; CI: 61%; content functional groups: 634 mol/g CNF–P: *w*: 4–5 nm ZP: −16.3 ± 1.6; CI: 45%; content functional groups: 1109 mol/g CNF–S: *w*: 4–5 nm ZP: −11.8 ± 0.6; CI: 56%; content functional groups: 444 mol/g	Ultrasonication (70% amplitude; 12 min, 20 KHz, 600 W)	50–500 μg/mL	Sterilised by autoclaving (20 min, 121 °C, 15 KPa)	No	24 h; 48 h	Cytotoxicity for carboxymethylated CNFs after 48 h at 500 μg/mL; bacteriostatic effect on *Escherichia coli* but not on *Lactobacillus reuteri*	[44]
Fibrillated Celluloses	Cytotoxicity (MTS), ROS, TEER, inflammation	Caco-2/HT29MTX/Raji B	Wood pulp	C20: *d*: 2.07 ± 0.03 μm; PDI: 0.871 ± 0.05; ZP: 46.4 ± 1.7 mV; C21: *d*: 1.24 ± 0.049 μm; PDI: 0.723 ± 0.132; ZP: −23.8 ± 1.83; C22: *d*: 2.46 ± 0.184 μm; PDI: 0.532 ± 0.25; ZP: −27.4 ± 6.98; C23: *d*: 1.449 ± 0.029 μm; PDI: 0.924 ± 0.083; ZP: −19.10 ± 0.93; C24: *d*: 0.809 ± 0.034 μm; PDI: 0.686 ± 0.036; ZP: −12.50 ± 0.83; C25: *d*: 0.646 ± 0.141 μm; PDI: 0.585 ± 0.208; ZP: −5.20 ± 0.19;	Homogenisation (Vortex-Genie 2, 10 min)	0.4% (*w*/*w*)	Metal impurities	Yes + lysosomal digestion	1, 6, 24, or 48 h	No effects	[163]
CNFs	Cytotoxicity (MTT)	HepG2	Sugarcane bagasse; TEMPO oxidation, sterilised, mechanical	*w*: 20 nm	NA	0.01–0.5 (*w*/*w*)	Sterilised before use following ISO10993-12	No	48 h	No cytotoxic effects	[164]
CNFs	Cytotoxicity (alamarBlue)	Caco-2	softwood kraft pulp (3% *w*/*w*, slurry form); ultrafine grinder	*L*: several hundred μm *w*: 50 nm ZP: −48 to −5 Mv	Homogenisation (vortex mixer)	0–500 μg/ml	NA	No	48 h	No cytotoxic effects	[145]
Multiple CNFs, carboxymethylated	Cytotoxicity (MTT)	Caco-2	Cotton linter pulp; mechanical stirring or carboxymethylated pretreatment; HPH	ZP: 12.4 ± 1.7, 21.8 ± 1.2, 26.7 ± 1.0, 34.2 ± 2.2 mV carboxyl content: 0, 0.36, 0.72, and 1.24 mmol/g	Vortex mixer	100–1000 μg/mL	Sterilised by filtration	No	24 h	No cytotoxic effects	[43]
mDTEB- CNF and CNFs	Cytotoxicity (LDH release; resazurin)	Caco-2/HT29MTX/Raji B	Wood pulp; mechanical treatment; autoclaved	mDTEB- CNF: *d*: 24.95 µm; ZP: −36.20 mV CNFs: *d*: 8.17 µm; ZP: −35.60 mV	NA	0.75% (*w*/*w*) and 1.5% (*w*/*w*)	Endotoxin levels using the EndoZyme^®^ recombinant factor C (rFC) assay; microbiological assessment	Yes	24 h	No cytotoxic effects	[51]
CNFs	Cytotoxicity (ATP, MTT; activations of caspases), oxidative stress (H_2_DCFA; glutathione; mtROS), lysosomal damage, release of IL-1β and TNF-α	Hepa 1–6 cell Kupffer cells (KUP5)	NA	CNFs-1: *L*: 6091 ± 2732 nm; *w*: 72.6 ± 63.6 nm; *L*/*d*: 83.4 ± 51.5; *d*: 5354.2 ± 1897.5; ZP: −12.3 ± 2 mV; CNFs-2: *L*: 6710 ± 5610 nm; *w*: 38.7 ± 33.4 nm; *L*/*d*: 172.1 ± 105.8; *d*: 5590.5 ± 3676.4; ZP: −11.0 ± 2.6 mV;	Vortexing + bath ultrasonication (15 min, 42 Khz, 100 W)	25–200 µg/mL	Endotoxin level using the limulus amebocyte lysate (LAL)	No	24 h	No cytotoxic effect in Hepa 1–6 cells; decreased viability and alterations in KUP5 cells (ATP assay); no induction of abiotic ROS and abiotic GSH	[158]
CNFs/TiO_2_	Cytotoxicity (MTT), toxicity to intestinal bacteria	Caco-2 FHC	Wood pulp; mechanical treatment followed by sonication	ZP: −36.50 ± 1.13 mV	NA	50–1000 µg/mL	NA	No		No cytotoxic effects; no effects on the growth *of Escherichia coli P-24*, *Lactobacillus acidophilus ADH*, and *Bifidobacterium animalis* Bif-6	[161]
CNFs/Ag	Cytotoxicity (MTT; WST-8 assays)	FHC Caco-2	CNF slurries	ZP: −23.03 ± 0.50 mV; size: *w*: 27.59 ± 10.53 nm	NA	50–1000 µg/mL	NA	No	24 h	Cytotoxic effects observed at higher concentrations	[162]

Notes: NA—not available; MTT—(3-(4,5-dimethylthiazol-2-yl)-2,5-diphenyltetrazolium bromide); MTS—(3-(4,5-dimethylthiazol-2-yl)-5-(3-carboxymethoxyphenyl)-2-(4-sulfophenyl)-2H tetrazolium); LDH—lactate dehydrogenase; ROS—reactive oxygen species; TEER—transepithelial electrical resistance; HTD—highest tolerated dose; mDTEB—meso-dichlorotriazineethyl BODIPY; FITC—fluorescein isothiocyanate; RBITC—rhodamine B isothiocyanate; N—submitted to chemicals and mechanical treatment; NM—not submitted to mechanical treatment; *L*—length (nm); *w*—width (nm); *d*—diameter (nm); *L*/*d*—aspect ratio; ZP—z-potential (mV); Y—yield (%); CI—crystallinity index; HPH—high-pressure homogenisation; AH—acid hydrolysis; S—sonication.

**Table 3:** Summary of in vivo toxicity studies after oral exposure to CNMs.

Tested CNM	In Vivo Model	Cellulose Source; Isolation Method: Isolation Conditions	Material Properties	Dispersion Method	Sterility or Endotoxin Assessment	Dose, Administration Route	Time of Exposure	Outcomes	Reference
CNCs	Crl:CD(SD)BR rats	NA	*L*: 92 ± 6 nm; *w*: 6.3 ± 0.4 nm *L*/*d*: 14.6; SSA: 399 ± 23 m^2^/g; average sulphur content: 0.73%	Ultrasonication (1000 J/10 mL)	NA	500, 1000, and 2000 mg/kg, oral gavage	14 days, single dose (OECD test guidelines 425)	No toxic effects observed (LD50 > 2000 mg/kg)	[159]
CNCs	Crl:CD(SD)BR rats	NA	*L*: 92 ± 6 nm; *w*: 6.3 ± 0.4 nm *L*/*d*: 14.6; SSA: 399 ± 23 m^2^/g; average sulphur content: 0.73%	Ultrasonication (1000 J/10 mL)	NA	500, 1000, and 2000 mg/kg, oral gavage	28 days, daily (OECD test guidelines 407)	No toxic effects observed ((NOEL > 2000 mg/kg/day). Normal neurological, body weight, weight gain, and food consumption parameters	[159]
CNCs	Crl:CD(SD)BR rats	NA	*L*: 92 ± 6 nm; *w*: 6.3 ± 0.4 nm *L*/*d*: 14.6; SSA: 399 ± 23 m^2^/g; average sulphur content: 0.73%	Ultrasonication (1000 J/10 mL)	NA	500, 1000, and 2000 mg/kg, oral gavage	? (OECD test guidelines 474)	No genotoxic effects. No micronuclei at a maximum tested dose of 2000 mg/kg	[159]
CNFs	*Rattus norvegicus albus*, male	Peach palm, NA	NA	NA	NA	7%, 14%, 21% (*w*/*w*), diet	30 days, daily	Increased weight over time. No effects on glycaemic rate, triglyceride levels, total cholesterol (blood), or mineral/nutrients loss (faeces). No hepatic damage (histological analysis)	[9]
BioPlus^®^ lignin- coated L-CNCs; BioPlus^®^ lignin- coated L-CNFs	Albino Sprague Dawley rats, female	NA	L-CNCs: *L*: 317 ± 60 nm; *w*: 14 nm; ZP: −18 mV, rod-shaped; average sulphur content: 0.05%; CI: 98% L-CNFs: *L*: 500 nm to several microns; *w*: 14–200 nm; ZP: −22 mV; average sulphur content: 0.09%; CI: 97%	Ultrasonication (10 min, Sonics VCX-750)	NA	5000 mg/kg, oral gavage	14 days, single dose (up and down procedure in rats, OPPTS 870.1100)	No acute oral toxicity	[172]
CNFs	Wistar Han rats, male	Softwood bleached kraft fibre, mechanical ultrafine friction grinding; autoclaved	*L*: 6710 ± 5611 nm; *d*: 64 ± 29 nm; *NtN*: 335.60 ± 232.66 nm; *L*/*d*: 107.6 ± 54.5; SSA: 34 m^2^/g	NA	Endotoxin levels using the EndoZyme^®^ recombinant factor C (rFC) assay; microbiological contaminations	1% (*w*/*w*), oral gavage		Postprandial rise in serum triglycerides reduced by 36%	[10] ^1^
CNFs	Wistar Han rats, male	Softwood bleached kraft fibre, mechanical ultrafine friction grinding; autoclaved	*L*: 6710 ± 5611 nm; *d*: 64 ± 29 nm; *NtN*: 335.60 ± 232.66 nm; *L*/*d*: 107.6 ± 54.5; SSA: 34 m^2^/g	NA	Endotoxin levels using the EndoZyme^®^ recombinant factor C (rFC) assay; microbiological contaminations	1% (*w*/*w*), oral gavage	35 days, twice a week	Reduction in weight gain (average 30–40% less weight), No damage to the liver, spleen, kidneys, and small intestine (histological analysis). No effect on hepatic, lipid, and renal markers; no alterations in electrolytes, blood cell counts, or haematological measurements	[62] ^1^
CNFs	Wistar Han rats, male	Softwood bleached kraft fibre, mechanical ultrafine friction grinding; autoclaved	*L*: 6710 ± 5611 nm; *d*: 64 ± 29 nm; *NtN*: 335.60 ± 232.66 nm; *L*/*d*: 107.6 ± 54.5; SSA: 34 m^2^/g	NA	Endotoxin levels using the EndoZyme^®^ recombinant factor C (rFC) assay; microbiological contaminations	1% (*w*/*w*), oral gavage (CNFs alone or in food matrix)	35 days, twice per week	Effects of CNF ingestion on bacterial genus and species diversity. Downregulation of claudins Cldn2 and Cldn3; gap-junction proteins Gja3 and Gjd2; integrins Itga2, Itga3, and Itgav; cadherins Cdh1; and adherens junction protein Nectin (Pvrl2)) in ileal mucosa. Upregulation of the claudin Cldn10 and cytokines IL-7, IL-18, IL-5, and IL-10 in the ileal mucosa	[122] ^1^
CNFs (fibrillated cellulose)	Albino Sprague Dawley rats	Wood pulp, mechanical homogenisation	*L*: 227.7 nm ± 103.3 μm *w*: 25.06 ± 6.29 nm *d*: 3330 ± 407 nm PI: 0.836 ± 0.190 ZP: −37.5 ± 1.67 mV	Homogenisation (Disruptor Genie 2, 10 min, 60 kHz; 240 W; 3000 rpm)	Assessment of impurities and microbiological contaminations	2, 3, or 4% (1044, 1550, and 2194 mg/kg/day and 1302, 1886, and 2667 mg/kg/day, for male and female rats, respectively)	90 consecutive days, repeated-dose exposure (OECD Test Guideline 408)	No toxicological effects. No-observed-adverse-effect level of 2194.2 mg/kg/day (males) and 2666.6 mg/kg/day (females). No observed clinical alterations in mortality, skin, fur, eyes, mucous membranes, secretions and excretions, body weight, food consumption, etc. No observed alterations in the cranial, thoracic, abdominal, and pelvic cavities, including associated organs and tissues. Histological vacuolisation of periportal hepatocytes (variably sized, clear cytoplasmic vacuoles) in the liver in the 4% CNCs and 4% cellulose groups, without hepatocyte degeneration or any other pathological observations. No alterations in haematological factors, serum chemistry, or urine parameters	[176]
CNCs	Albino Sprague Dawley rats	Wood pulp; AH (H_2_SO_4_)	*L*: 25 to 250 nm; *w*: <10 nm; *d*: 893 ± 251 nm; PDI: 0.51 ± 0.02; ZP: −50.8 ± 6 mV, rod-shaped	Homogenisation (Vortex Genie 2, 10 min)	Assessment of impurities and microbiological contaminations	2, 3, and 4 *w*/*w*%, diet (1056, 1584, and 2085 mg/kg/day and 1278, 1930, and 2683 mg/kg/day for the male and female rats, respectively)	Pilot test: 7 and 14 days, daily (OECD Test Guideline 407) 90 consecutive days, repeated-dose exposure (OECD Test Guideline 408)	Pilot test: No adverse effects associated with feeding 5% CNCs over 7 days or up to 1.2% CNCs over 14 days. 90 consecutive days test: No toxicological effects. No-observed-adverse-effect level of 2085.3 (males) and 2682.8 (females) mg/kg/day. No observed clinical changes in mortality, skin, fur, eyes, mucous membranes, secretions, or excretions. Increased body weight and food consumption in female rats fed with 4% CNCs, with increased heart, liver and spleen weight Vacuolisation of periportal hepatocytes (variably sized, clear cytoplasmic vacuoles) in the liver in the 4% CNCs and 4% cellulose groups, without hepatocyte degeneration or any other pathological observations. No observed histological alterations in the other analysed organs (including the colon, kidneys, stomach, spleen, ileum with Peyer’s patches, jejunum, brain, reproductive organs, eyes, etc.). No alterations in haematological factors, serum chemistry, or urine parameters	[138]
CNFs	C57BL/6 mice	Softwood kraft pulp, ultrafine grinder	*L*: several hundred μm *w*: 50 nm ZP: −48 to −5 Mv	Homogenisation (vortex mixer)	NA	30 mg/kg BW/day, oral gavage	4–6 weeks	No toxicological effects. Dysregulated glucose homeostasis, decreased lean body mass (male), no differences in food consumption, no histological damage to the small intestine	[145]
CNCs modified with oxalate esters	Albino Sprague Dawley rats	Cotton seeds; AH (H_2_SO_4_)	Average particle size: 100 nm ZP: −50 mV to −10 mV	NA	NA	50 and 100 mg/kg	7 days, daily	Hepatic injury (100 mg/kg). Normal liver weight, alkaline phosphatase activity, and lipid peroxidation; increased aspartate aminotransferase, alanine aminotransferase, and myeloperoxidase activities; increased nitric oxide synthase, Bcl-2-associated X protein, and MPO (liver). No effect on SOD, GSH, H_2_O_2_, NO, and MDA; decreased CAT and GPx	[59]
Multiple CNFs, carboxymethylated	KM mice, female	Cotton linter pulp, mechanical stirring or carboxymethylated pretreatment; HPH	ZP: 12.4 ± 1.7, 21.8 ± 1.2, 26.7 ± 1.0, 34.2 ± 2.2 mV carboxyl content: 0, 0.36, 0.72, and 1.24 mmol/g	Homogenisation (vortex mixer)	Sterilised by filtration	1% or 3.5% (*w*/*w*), oral gavage	8 weeks, daily	No toxicological effects. Decreased body weight. No alterations in haematological measurements or serum lipid, hepatic, renal, and heart markers	[43]
R-CNC_sulf_ R-CNC_AC_ D-CNC_Ac_	Mice, male and female	Cotton microcrystalline cellulose (R-CNC_sulf:_ AH, 65 wt% H_2_SO_4_ 1:20, 45 °C, 30–90 min; R-CNC_AC_ and D-CNC_Ac:_ acetylated surface obtained via solvolysis in the acetic acid/phosphotungstic acid system)	R-CNC_sulf_: *L*: 195 ± 30 nm; *d*: 234 ± 3 nm; ZP: −53 ± 2 45 mV; H: 9.5 ± 2.0 nm R-CNC_AC_: *L*: 165 ± 35 nm; *d*: 200 ± 10 nm; ZP: −45 ± 6 mV; H = 8.5 ± 2.0 nm D-CNC_Ac_: *d*: 110 ± 15 nm; ZP: −38 ± 4 mV; H: 3–12 nm	NA	Hydrosols filtered and treated with ultraviolet	2000 mg/Kg, oral gavage	14 days, single dose (OECD test guidelines 425)	No toxicological effects. No morphological changes of the liver, kidneys, heart, and spleen	[55]
CNCs; cationic CNCs	Murine models with chronic renal failure and hyperphosphatemia	Softwood bleached kraft pulp, AH (H_2_SO_4_); cationisation agent (EPTAC) in ultrasonic bath	Cationic CNCs: *L*: 150 nm *d*: 10 nm; ZP: 62.3 mV CNCs: ZP: −31.6 mV;	NA	NA	0.6% (*w*/*w*), oral gavage	14 days, daily	Elevated aspartate aminotransferase, cortical and cerebellar glutathione, and lipid peroxidation levels	[170]
CNCs	Albino Sprague Dawley rats, male	P. longifolia seeds; HCL followed by ultrasonication	*d*: 5–70 nm	NA	NA	50 and 100 mg/kg BW, oral gavage	14 days, daily	Elevated aspartate aminotransferase, cortical and cerebellar glutathione, and lipid peroxidation levels; no altered histology of neurons, hippocampus, and Purkinje layers; no alterations of body and organ weights, albumin, cortical and cerebellar catalase, and glutathione S-transferase levels	[171]
CNFs	C57BL/6 mice, female (inflammatory bowel disease model)	Wood, adlay (C. lacryma-jobi) chaff, and hijiki seaweed (Sargassum fusiforme); mechanical treatments	NA	NA	NA	0.1% (*w*/*w*), via drinking water	5 days, ad libitum	Colon lengths of CNFs obtained from adlay and hijiki seaweed were longer than controls and CNFs obtained from wood. In the colon of control CNFs and those obtained from wood, histological alterations were observed (i.e., erosion, shortening, or destruction of the crypts, and oedema), which were ameliorated by the other CNFs. Reduction in myeloperoxidase and NF-κB staining after exposure to CNFs obtained from adlay and hijiki seaweed compared to CNFs obtained from wood and control CNFs	[177]
CNFs	C57BL/6 mice, female (inflammatory bowel disease model)	Japanese pear and wood; mechanical treatments	NA	NA	NA	5.7% (*w*/*w*) for CNFs from Japanese pears, 1% for CNFs from wood, via drinking water	5 days, ad libitum	Colon lengths of CNFs obtained from pears were longer than those of controls and CNFs obtained from wood. In the colon of control CNFs and those obtained from wood, histological alterations were observed (e.g., erosion, shortening, or destruction of the crypts, and oedema), which were ameliorated by the other CNFs. Reduction in myeloperoxidase, collagen deposition, and NF-κB staining after exposure to CNFs obtained from pears and seaweed compared to those obtained from wood and controls	[178]
CNCs	C57BL/6 mice	NA	NA	NA	NA	0.1 and 0.2% (*w*/*w*), via drinking water	7 weeks, ad libitum	Administration of 0.2% CNCs decreased the relative abundance of Streptococcaceae and Rikenellaceae, and increased that of Lactobacillaceae; weight gain suppression in high-fat-diet-fed mice treated with 0.2% CN, with lower accumulation of epididymal and subcutaneous fat; no differences in levels of fasting blood glucose and glucose tolerance	[175]
CNCs	Healthy donors’ faecal matter; Wistar rats, male	Microcrystalline cellulose, AH (H2SO4) followed by ultrasonication	CNCs1: *L*: 346.28 ± 102.12 nm; *L*/*d*: 26.16; *d*: 367 ± 89 nm (DLS); *d*: 13.23 ± 0.84 nm (AFM); SSA: 8.28 m^2^/g CNCs2: *L*: 276.58 ± 135.22 nm; *L*/*d*: 32.77; *d*: 230 ± 68 nm (DLS); *d*: 8.44 ± 0.75 nm (AFM); SSA: 18.33 m^2^/g; CNCs3: *L*: 125.01 ± 25.43 nm; *L*/*d*: 38.58; *d*: 104 ± 25 nm (DLS); *d*: 3.24 ± 0.75 nm; SSA: 27.9 m^2^/g	NA	NA	250 mg/kg BW, oral gavage	14 days, twice per day	Size-dependent increase in short-chain fatty acids (including acetate, butyrate, and propionate); increased *Bifidobacterium*	[173]

Notes: NA—not available; CNCs—cellulose nanocrystals; CNF—cellulose nanofibres; *L*—length (nm); y—width (nm); *NtN*— node-to-node length, defined as the distance between the centres of two nodes; H—height (nm); *d*—diameter (nm); PI—polydispersity index; *L*/*d*—aspect ratio; ZP—z-potential (mV); Y—yield (%); CI—crystallinity index; SSA—specific surface area; HPH—high-pressure homogenisation; AH—acid hydrolysis; S—ultrasonication; DLS—dynamic light scattering; AFM—atomic force microscopy. ^1^ Same experiment.

**Table 4:** Issues to consider when addressing the toxicity assessment of NMs/CNMs.

Specific Issues in the Toxicology Assessment of CNMs
Chemical impurities
Presence of biological contaminants (e.g., endotoxins)
Physicochemical characteristics in cellular moieties
Dispersion and stability of CNMs in biological media

## Data Availability

Not applicable.
